# SNP genotyping reveals substructuring in weakly differentiated populations of Atlantic cod (*Gadus morhua*) from diverse environments in the Baltic Sea

**DOI:** 10.1038/s41598-020-66518-4

**Published:** 2020-06-16

**Authors:** Roman Wenne, Rafał Bernaś, Agnieszka Kijewska, Anita Poćwierz-Kotus, Jakob Strand, Christoph Petereit, Kęstas Plauška, Ivo Sics, Mariann Árnyasi, Matthew P. Kent

**Affiliations:** 1 Institute of Oceanology, Polish Academy of Sciences, Powstańców Warszawy 55, 81-712 Sopot, Poland; 2 Department of Migratory Fishes in Rutki, Inland Fisheries Institute, Olsztyn, 10-719 Poland; 3 Arctic Research Centre, Department of Bioscience, Aarhus University, Frederiksborgvej 399, 4000 Roskilde, Denmark; 4 GEOMAR, Helmholtz Centre for Ocean Research Kiel, Research Division 3: Marine Ecology, Research Unit: Evolutionary Ecology of Marine Fishes, Düsternbrooker Weg 20, 24105 Kiel, Germany; 5 Bruno-Lorenzen-Schule Schleswig, Spielkoppel 6, 24837 Schleswig, Germany; 6 Fisheries Service under the Ministry of Agriculture Division of Fisheries Research & Science, Smiltynes 1, 91001 Klaipeda, Lithuania; 7 Institute of Food Safety, Animal Health and Environment “BIOR”, Riga, Latvia; 8 Centre for Integrative Genetics (CIGENE), Department of Animal and Aquacultural Sciences (IHA), Faculty of Life Sciences (BIOVIT), Norwegian University of Life Sciences (NMBU), PO Box, 5003 Aas, Norway

**Keywords:** Evolutionary biology, Marine biology

## Abstract

Atlantic cod (*Gadus morhua*) is one of the most important fish species in northern Europe for several reasons including its predator status in marine ecosystems, its historical role in fisheries, its potential in aquaculture and its strong public profile. However, due to over-exploitation in the North Atlantic and changes in the ecosystem, many cod populations have been reduced in size and genetic diversity. Cod populations in the Baltic Proper, Kattegat and North Sea have been analyzed using a species specific single nucleotide polymorphism (SNP) array. Using a subset of 8,706 SNPs, moderate genetic differences were found between subdivisions in three traditionally delineated cod management stocks: Kattegat, western and eastern Baltic. However, an *F*_ST_ measure of population differentiation based on allele frequencies from 588 outlier loci for 2 population groups, one including 5 western and the other 4 eastern Baltic populations, indicated high genetic differentiation. In this paper, differentiation has been demonstrated not only between, but also within western and eastern Baltic cod stocks for the first time, with salinity appearing to be the most important environmental factor influencing the maintenance of cod population divergence between the western and eastern Baltic Sea.

## Introduction

Sustainable exploitation of living marine resources by fishery, aquaculture and biotechnology, and monitoring and predicting the effects of climate changes require an understanding of taxonomy and population biology. Populations are sustainably exploited if the removal of individuals does not reduce the ability of a population to reproduce and maintain its phenotypic and genetic diversity. Such populations have been defined for conservation purposes as “evolutionary significant units”^1^, and traditionally have been defined using genetic methods such as analyses of allozymes, nuclear DNA loci, microsatellites and mitochondrial DNA^2^ and knowledge of fish biology and morphology^3^. The management units are defined for reporting on stock assessment and catches by different countries. The issue of inconsistency between existing management units and population biology and genetic differentiation has been reported for some marine fish species^4–6^. Presently, population genetic analysis using classical genetic markers is being replaced by more detailed genomic analysis which provides qualitatively new information on stock differentiation and identification^7–14^. Specific methods include genome-wide genotyping using a large number of single nucleotide polymorphisms (SNPs) and next-generation sequencing (NGS). NGS for population analyses is a powerful tool that arguably provides the greatest insight into population genomics but can be expensive and demands significant data analysis. In contrast, genotype data for relatively many SNPs can be generated quickly for large numbers of individuals using genotyping arrays and raw data requires little pre-processing before it can be analyzed^15^. A large number of SNP loci in comparison with few genetic markers are better able to report subtle differences in genomic variation and their robustness is an advantage in evolutionary and population biology studies including exploited species with high dispersal potential in the oceans^16–18^. Additionally SNPs may affect protein function and expression levels directly and hence are subject to evolutionary selective forces^19^. Genotyping a significant number of SNP loci also provides an opportunity to identify ‘outliers’ (i.e. loci under selection^20,21^), which can be more informative markers in defining conservation units in comparison with neutral SNP markers^22–26^.

Compared to many other harvested and aquacultured fish species, Atlantic cod has been subject to extensive population genetics analysis^4,27,28^, that have provided insight into the links between genomics, biology and life-history. For example, studies using neutral molecular markers, such as microsatellites, have detected very weak differentiation between populations of cod^29^, whereas loci under selection from environmental conditions as temperature, salinity and depth, often display much stronger differentiation^30–32^. SNP analysis revealed diversity between eastern and western Atlantic and Baltic cod populations^33–36^, and using the same technology divergence within a genomic region between migrating and stationary ecotypes has been found despite high connectivity^37–41^. Genomic rearrangements (e.g. inversions of chromosome fragments) in cod populations differing in ecological behaviour, such as migration routes have been found^42^, and a subset of the SNP loci analyzed in this study has been reported as significantly correlated with temperature in North Atlantic cod populations^43,44^. It has been shown that despite mixing and migrations between the northeast Arctic cod and the Norwegian coastal cod populations, genomic islands undergo selection and reduced recombination, which promote divergence of these populations despite habitats overlap^45^. Finally, frequencies of some SNPs have been shown to be correlated with salinity in Baltic herring populations^46^.

The Baltic was transformed from a freshwater lake receiving melting ice waters into today’s brackish water sea body over 7000 years ago. It was colonized by a variety of marine species populations^47,48^, which could tolerate salinity as low as 5–7 ppt, including Atlantic cod. This species adapted to living in low salinity waters of the Baltic Sea despite requiring salinity above 12–14 ppt^13,49–53^ for successful spawning^54,55^. For the fisheries purposes, the Baltic cod is assessed and managed as western and eastern stocks, located in ICES Subdivisions 22–24 and 24–32 respectively^56^.

Significant transport of cod larvae from the North Sea to Skagerrak and Kattegat^57,58^ and mixing of western and eastern stocks of Baltic cod in the Arkona Basin (ICES subdivision 24) have been reported^22,59–62^. The dynamic changes of salinity and oxygenation in the Danish Straits and the open waters of Baltic Sea undoubtedly affect the condition and genetic divergence of the cod stocks in this area. Western populations of the Baltic cod may hybridize with the North Sea cod, as was suggested by Nielsen *et al*.^29^. Besides the important physiological differences between western and eastern Baltic cod such as haemoglobin polymorphisms^63,64^, genetic structure differences were reported at the level of population markers, e.g. microsatellites, *Pan* I locus, mtDNA^29^^,^^65–67^ and using SNP analysis^62,68,69^. Implications of population genetics structure in Baltic populations for management, have been summarized by Östman *et al*.^6^ and Wennerström *et al*.^70,71^.

Two main cod spawning areas in the Kattegat are situated along the Swedish coast. Western Baltic cod spawning areas include Sound, Kiel and Mecklenburg Bays and Arkona Basin^56,72^. Eastern Baltic cod spawning regions were Bornholm Basin, Słupsk Furrow, Gdańsk Deep and Gotland Basin in Southern Baltic. The main area of spawning is the Bornholm Basin where the eastern Baltic cod migrated every season^62,73–75^. Due to reduced inflow of North Sea water into the Baltic Proper and other changes in environmental and ecological conditions (e.g. oxygen deficiency, low nutrition, infestation with parasites, increased water temperature, size selective fishing), eastern Baltic cod underwent changes in the biology; slower growth rate and maturation at a smaller size^76,77^. The anoxic or hypoxic conditions in the Baltic Sea, exceptionally pronounced in recent years inflicted contraction of southern populations^77^, and limited the size of the reproductive volume of eastern Baltic cod^54^. In recognition of a serious threat to the eastern Baltic cod stock, fishing for cod in ICES subdivisions 24, 25 and 26 has been banned by the European Commission in 2019, and restricted beginning on 1^st^ January 2020.

Genetic differentiation at functionally important genes between cod stocks in the north-western Atlantic have been related to local adaptations caused by differential selection pressure among spawning aggregations^14^. In the Baltic, differences in adaptation of western and eastern cod stocks to low salinity have been described^13,52,69^. SNP array has been used for the first time to characterise different stocks in the Baltic. Earlier population genetic studies conducted with this technique included only limited number of individuals or few sampling locations inside Baltic. However, to date no genetic differentiation within sub-stocks of western and eastern Baltic cod populations have been reported. The aim of this research was to characterize for the first time differences in structure of sub-stocks within eastern and western Baltic cod populations and the transition zone with the North Sea using a large number of SNP loci. The possible existence of sub-structuring of the eastern stock within a management unit is indicated.

## Results

### Genetic diversity

**Figure 1:**
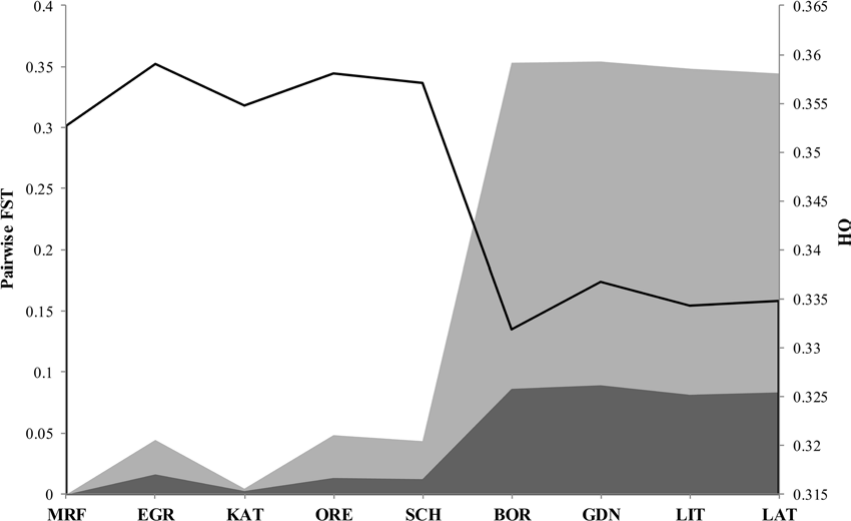
Pairwise *F*_ST_ values calculated according to the westernmost sample (MRF, Scotland) for all 8076 loci (dark grey) and 588 outlier loci (light grey). Black line represents observed heterozygosity (H_o_) across West-East transect.

**Figure 2:**
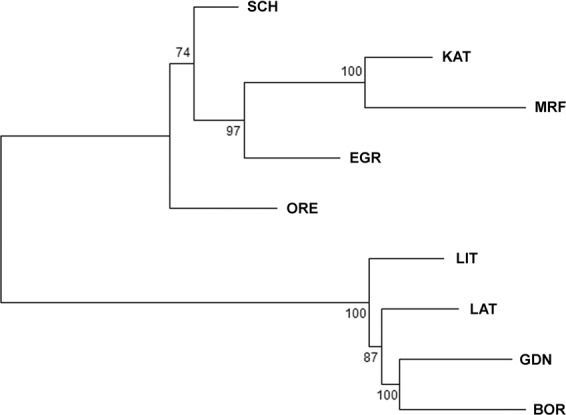
A neighbor-joining tree constructed using Nei’s distances among the nine cod populations. Bootstrap probabilities are shown on the tree.

In total 8076 SNPs that passed quality and informativity control were used to estimate the genetic diversity of cod populations from the Baltic and North Sea. The overall inbreeding coefficient *F*_IS_ obtained by AMOVA was very low (−0.0032) and statistically insignificant (p = 0.62). The global *F*_ST_ across all nine populations was 0.0396 (p < 0.01), which indicates a moderate^78^ level of differentiation. Low, but statistically significant levels of differentiation were found between pairs of samples from North Sea (EGR), Kattegat (KAT) and west Baltic (SCH). There was no statistically significant differentiation for the pair from the Belt Sea (SCH) and Øresund (ORE) in western Baltic. While the greatest divergence, above 0.08, was observed between Scotland (MRF) and four populations from the eastern Baltic (GDN, BOR, LAT and LIT) (Fig. 1; Table 1). *F*_ST_ values between western samples (SCH, ORE, KAT and EGR) and samples from the eastern Baltic were at a similar level: from 0.0510 for the pair LIT-SCH to 0.0721 for the pair GDN-KAT (Table 1). An Neighbour Joining tree showing corrected *F*_ST_ distance was constructed within all nine cod populations from the 8076 SNP data set (Fig. 2). Western samples (KAT, MRF, EGR, SCH and ORE) were shown to form one branch of the tree, while four populations from the East Baltic (LAT, LIT, GDN, BOR) formed a separate clade; all clades had a high value of bootstrap reliability. The highest values of observed heterozygosity (Table 2) were in western samples (0.353 in MRF to 0.359 in EGR). The heterozygosity levels in the east Baltic samples (LAT, LIT, GDN and BOR) were lower and ranged from 0.332 in BOR to 0.337 in GDN. The vast majority of loci were in Hardy-Weinberg equilibrium (HWE) in all populations, the greatest fraction of SNPs with HWE departure (p < 0.05) were observed in populations from Egersund (EGR; 507 polymorphic sites) and the lowest in GDN and KAT (174 and 175 respectively) (Table 2).

**Table 1:** Below diagonal: pairwise *F*_ST_ values based upon 8076 polymorphic SNPs in 9 sampled populations of cod, calculated in Arlequin.

	LAT	LIT	GDN	BOR	SCH	ORE	KAT	EGR	MRF
LAT	*2695.52*	NS	NS	NS	*	*	*	*	*
LIT	0.0000	*2689.20*	NS	NS	*	*	*	*	*
GDN	0.0011	0.0012	*2705.44*	NS	*	*	*	*	*
BOR	0.0012	0.0010	0.0000	*2693.28*	*	*	*	*	*
SCH	0.0518	0.0510	0.0569	0.0557	*2875.41*	NS	*	*	*
ORE	0.0527	0.0519	0.0564	0.0553	0.0000	*2864.10*	*	*	*
KAT	0.0685	0.0678	0.0721	0.0702	0.0053	0.0060	*2893.11*	*	*
EGR	0.0588	0.0578	0.0614	0.0611	0.0061	0.0077	0.0086	*2895.84*	*
MRF	0.0829	0.0810	0.0895	0.0865	0.0118	0.0127	0.0024	0.0163	*2774.74*

All values being significant for p = 0.05 are indicated as * and non-significant as NS. On diagonal: average number of pairwise difference within population.

**Table 2:** Genetic parameters of the 9 cod *Gadus morhua* sampled populations.

Sample	n	NPL	MNA	H_O_	H_E_	DHWE	BC	*F*_IS_
LAT	30	7754	1.963	0.3347	0.3341	210	11	−0.0005
LIT	30	7730	1.962	0.3343	0.3344	207	13	−0.0036
GDN	24	7657	1.95	0.3367	0.3356	174	2	−0.0044
BOR	21	7641	1.948	0.3319	0.3340	210	4	0.0057
SCH	30	7973	1.99	0.3571	0.3566	196	11	−0.0022
ORE	21	7895	1.98	0.3581	0.3553	192	2	−0.0090
KAT	23	7899	1.98	0.3548	0.3588	175	8	0.0107
EGR	27	7921	1.984	0.3590	0.3591	507	11	−0.0006
MRF	34	7886	1.983	0.3527	0.3489	239	13	−0.0188

Sample name, number of individuals **n**, number of polymorphic loci **NPL**, mean number of alleles **MNA**, observed and expected heterozygosity, loci deviating from HWE, after Bonferroni correction and population specific *F*_IS_. Significance at the p < 0.05 level.

Genetic relationships among cod populations and possible genetic admixture was calculated using the Bayesian algorithm in STRUCTURE. When a full set of 8076 polymorphic SNPs was used for all nine cod samples, the most probable number of populations was 2 (ΔK = 1329.3), with four samples from the east Baltic (LAT, LIT, GDN, BOR) distinguishing themselves from the remaining populations. The *F*_ST_ calculations based on 588 outlier loci (Supplementary information Table S1) for 2 groups, including 5 western (North Sea, Kattegat west Baltic) and 4 eastern Baltic samples, increased and indicated high genetic differentiation (0.187, p < 0.001) (Table 3). *F*_ST_ pairwise comparisons between the East Baltic samples (LIT, LAT, GDN and BOR) remained non-significant, while *F*_ST_ for pair SCH-ORE became statistically significant. Pairwise *F*_ST_ values between remaining populations increased significantly and generally reproduced mapping of *F*_ST_ relations between samples.

**Table 3:** Below diagonal: pairwise *F*_ST_ values based upon 588 outliers SNPs in 9 sampled populations of cod, calculated in Arlequin.

	LAT	LIT	GDN	BOR	SCH	ORE	KAT	EGR	MRF
LAT	*206.71*	NS	NS	NS	*	*	*	*	*
LIT	0.0000	*204.76*	NS	NS	*	*	*	*	*
GDN	0.0019	0.0011	*204.43*	NS	*	*	*	*	*
BOR	0.0011	0.0028	0.0000	*204.21*	*	*	*	*	*
SCH	0.2402	0.2445	0.2500	0.2454	*229.91*	*	*	*	*
ORE	0.2456	0.2507	0.2523	0.2475	0.0037	*229.85*	*	*	*
KAT	0.3132	0.3174	0.3225	0.3202	0.0226	0.0236	*211.67*	*	*
EGR	0.2492	0.2526	0.2582	0.2550	0.0145	0.0234	0.0264	*231.90*	*
MRF	0.3443	0.3484	0.3544	0.3533	0.0431	0.0486	0.0041	0.0446	*197.77*

All values being significant for p = 0.05 are indicated as * and non-significant as NS. On diagonal: average number of pairwise difference within population.

**Figure 3:**
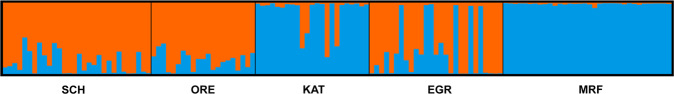
Estimated *Gadus morhua* population structure by STRUCTURE software based on 175 outlier loci in the samples from North Sea, Kattegat and West Baltic.

**Figure 4:**
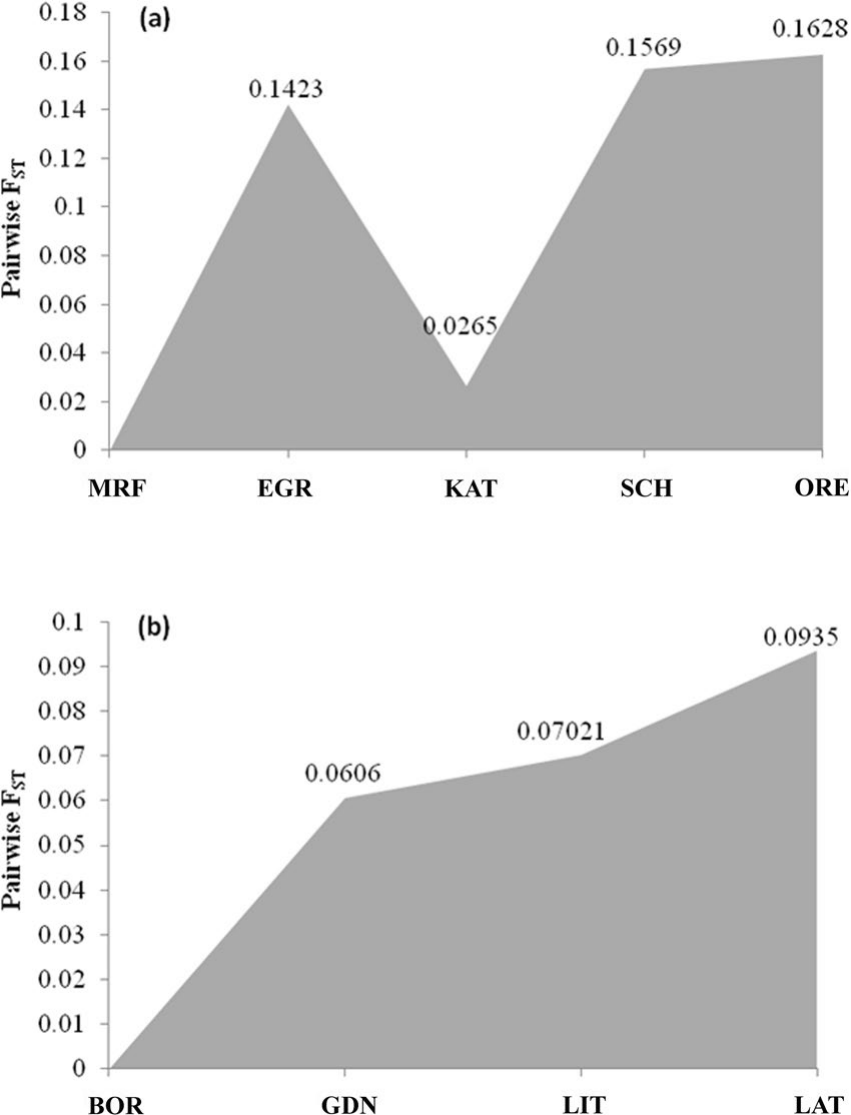
Pairwise differences (*F*_ST_) between: (**a**) samples from North Sea, Kattegat and West Baltic based on 175 outlier loci and (**b**) samples from eastern Baltic stock based on 89 outlier loci.

**Figure 5:**
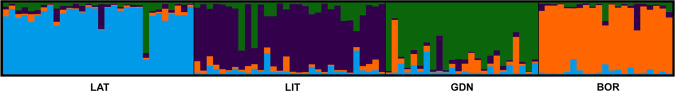
Graph represents the STRUCTURE results for the Baltic dataset, based on 89 outlier loci and best K = 4.

**Figure 6:**
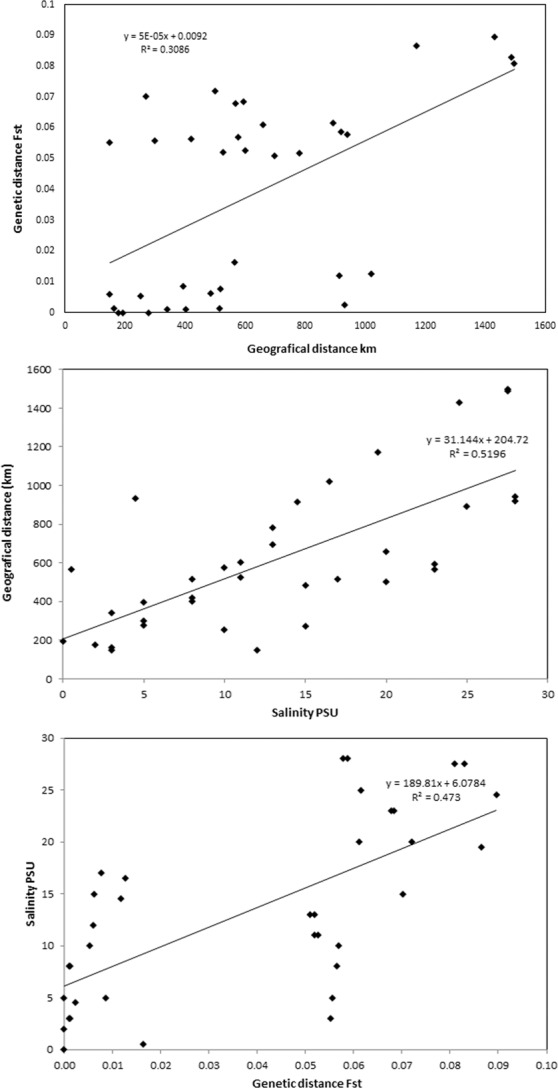
Relationships between geographical and genetic distance *F*_ST_ (upper graph), bottom salinity and geographical distance (middle graph), genetic distance and bottom salinity (lower graph). P values from top: 0.001, 0.001 and 0.01.

The five samples from western Baltic, Kattegat and the North Sea were analyzed with a set of 175 outlier loci (Supplementary information Table S2). The maximum value of ΔK (279.4) was found for K = 2 and 2 clusters were identified capturing SCH + ORE + EGR, and KAT + MRF (Fig. 3), this distribution of samples does not coincide with their geographic origin. The sample from Kattegat was closely related to the Moray Firth sample while cod from the Egersund was grouping with samples from the Schlei (Belt Sea) and Øresund. The variation among groups was 10.04% while among individuals within populations only 0.14%. Pairwise differences were statistically significant (p < 0.001) and their value ranged from 0.012 for pair ORE – SCH to 0.162 for pair MRF – ORE. Pairwise differences between samples were similar to those observed in relations among the western samples based on outlier loci for all samples (Fig. 4a; Table 3). The four samples from the eastern Baltic stock (LAT, LIT, GDN, BOR) were analyzed with a set of 89 outlier *loci* (Supplementary information Table S3) and formed 4 clusters (ΔK = 90.98) (Fig. 5). Pairwise differences between samples correlated with geographic collocation of samples confirmed by Mantel test (Fig. 6). The lowest value of *F*_ST_ was observed among LAT (Latvia) and LIT (Lithuania) samples (0.05614, P < 0.000). The highest difference was noted for the pair BOR (Bornholm) – LAT (0.09358, P < 0.000) and intermediate values for other pairs of samples. Pairwise differences (*F*_ST_) values suggest some difference between the BOR sample and the remaining samples from the Baltic Sea (Fig. 4b). Three eastern most samples from the Baltic Sea formed 3 clusters (ΔK = 221.30) in the Structure analysis with the 76 outliers (Supplementary Information Fig. S1; Table S4). The fixation index calculated for the 3 samples indicated moderate differentiation (*F*_ST_ = 0.07552, p < 0.000). Pairwise *F*_ST_ distances were generally close and reached 0.06912 for LAT – GDN pair, 0.07044 for LIT – GDN and 0.08387 between LAT – LIT.

### Genetic distance and assignment test

**Figure 7:**
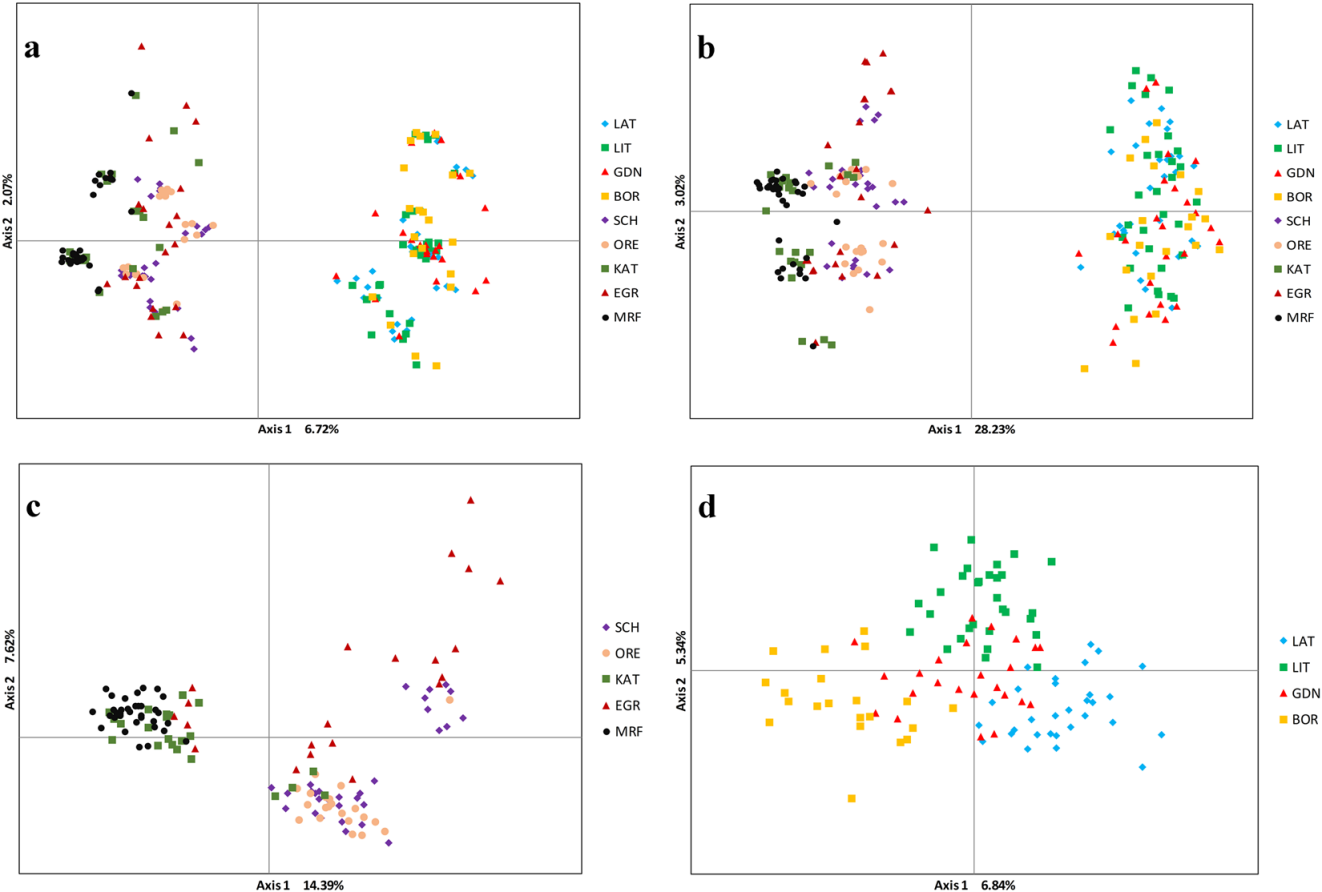
Principal Coordinates Analysis (PCoA) 2D plots imaging variation between all and outlier loci calculated for: (**a**) all samples with full set 8076 and (**b**) 588 outlier SNPs, (**c**) western samples - 175 outlier SNPs, (**d**) eastern Baltic stock samples - 89 outlier SNPs.

Principal coordinates analysis (PcoA) performed for the full marker set (8076 SNPs) showed low values of percentage of variation between axes, however the potential clades are well separated (Fig. 7). Analysis performed for 588 outlier loci revealed higher genetic variation of the 1^st^ axis and low differentiation on the 2^nd^ and 3^rd^ axes. For the western samples PcoA revealed the highest variation on the 1^st^ axis while lower on the 2^nd^, and, on the 3^rd^ axis only 3.70%. In the subset of eastern Baltic populations values were lower and for the 1^st^ axis reached 6.84% and respectively 5.34% and 4.79% of variability (Fig. 7).

**Figure 8:**
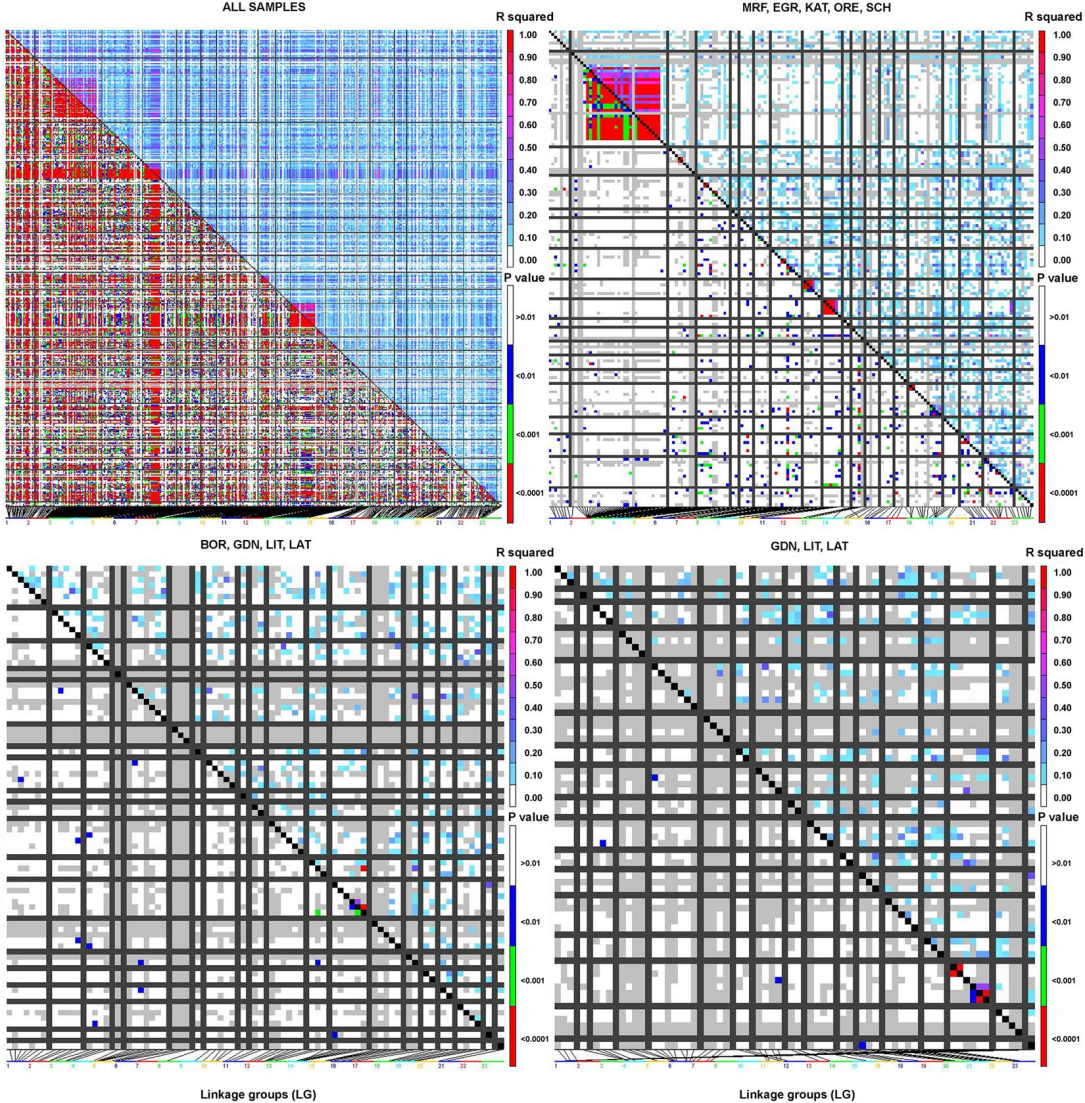
Heat maps of pairwise linkage disequilibrium (LD) values (r^2^) throughout the Atlantic cod genome constructed for four outlier loci subsets. Markers were ordered on the x and y axes based on genomic location so that each cell of the heat map represents a single marker pair located on particular chromosome. The r^2^ values for each marker pair are on the upper half of the heat map. The p values of each r^2^ estimate are on the bottom half of the heat map.

**Figure 9:**
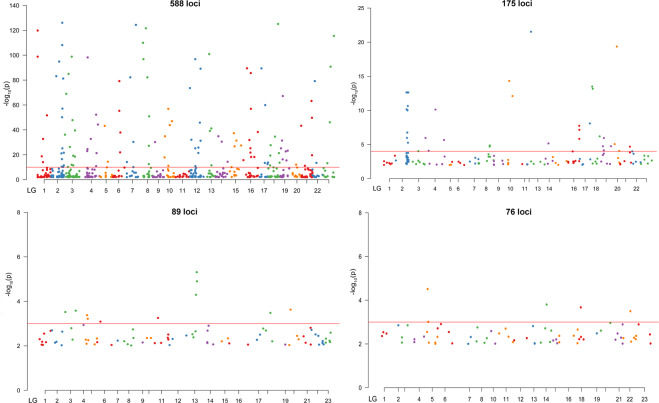
Manhattan plots of outlier analyses based on median log10(p value). The SNPs are distributed according to LG and their position within the LGs along the X axis^124,125^. The solid line is standard p value cutoff^124^.

To determine the most likely origin of all 240 cod individuals, assignment tests were conducted with the allele frequency based method that allowed the identification of potential migrants and estimated sample heterogeneity. In the west group of samples, the frequency of self-assignments varied from 30% for the KAT sample to 100% for the ORE. In the eastern group, values were much lower and ranged from 5% for BOR to 41% in LAT. Generally, only 48% of individuals were assigned to the population they were collected from with mean for the western group at 68% and 24% for the eastern. No genotypes of the individuals from the east Baltic group were represented in the Danish Straits/North Sea group and vice versa (Table 4). The Mantel test was significant for all applied comparisons with p values 0.001 for *F*_ST_ vs. geographic distance and geographic distance vs. bottom salinity and p = 0.01 for *F*_ST_ vs. bottom salinity (Fig. 6). Analysis of pairwise linkage disequilibrium (LD) based on 588 outlier loci for all investigated populations show 11 114 highly significant pairwise LDs. From that number 60.4% were inter-chromosomal LDs and respectively 39.6% intra-chromosomal. The largest block was revealed on linkage group LG2 and covered 22% of all LDs (Fig. 8). The smaller blocks were primarily on LG3, LG4 and LG5 and covered 9.6, 8.6 and 8.5 percent of all LDs. The share of found LDs decreased then to chromosome 23, where their share was only 1%, however they occurred on every LG. Calculations performed on the western dataset only for 175 detected outliers showed 389 highly significant LDs. The majority of them constituted intra-chromosomal LDs, 78.4%. The major and largest blocks were located on LG2 and 69% of all LDs belonged to him (Fig. 9). Next analysis, based on Baltic dataset and 89 outliers showed only two LDs, one inter and one intra-chromosomal, both related with LG13 (Fig. 8). The last analysed dataset containing three easternmost Baltic samples calculated with 76 detected outliers also show only two highly significant LDs, however what is important they were inter-chromosomal and located on LG16. Detailed description of LDs detected for datasets based on 89 outliers (BOR, GDN, LIT, LAT) revealed that they concerned loci ss1712298167 vs. ss1712303712 both located on scaffold 07407 (LG13) and ss1712298916 (scaffold 08672, LG12) vs. ss1712298167. LDs detected for easternmost samples (GDN, LIT, LAT) occurred for loci ss1712298846 vs. ss1712298845 both located on scaffold 08549 and for loci ss1712299176 (scaffold 09117) vs. ss1712297964 (scaffold 07099). All of them were on LG16. The distribution of the outlier loci across LGs are displayed on Manhattan plots constructed for same datasets (Fig. 9). The presented patterns of outlier loci locations are congruent with distribution of the detected LDs. Outlier subsets with detailed positions and gene annotations have been presented in Supplementary information Table S5.

**Table 4:** Results of the assignment test performed for 9 populations with 8076 loci, computed using GeneClass software.

	LAT	LIT	GDN	BOR	SCH	ORE	KAT	EGR	MRF
LAT	**40.82**	36.97	18.65	3.54	—	—	—	—	—
LIT	50.2	**26.29**	10.18	13.31	—	—	—	—	—
GDN	34.67	37.52	**23.64**	4.16	—	—	—	—	—
BOR	36.24	30.85	28.13	**4.75**	—	—	—	—	—
SCH	—	—	—	—	**77.59**	16.74	3.29	2.32	0.04
ORE	—	—	—	—	—	**100**	—	—	—
KAT	—	—	—	—	17.39	—	**30.43**	—	52.17
EGR	—	—	—	—	40.74	—	11.11	**44.45**	3.7
MRF	—	—	—	—	—	—	11.76	—	**88.23**

Individuals were assigned to the populations in which the genotype is most probable to occur. Values are given in percent. Self-assignment is indicated in bold.

## Discussion

The status of Atlantic cod in the Baltic Sea has been reported as an example of a geographically and genetically separated marginal subpopulation^79,80^. Populations of cod inhabiting the Baltic Sea have evolved differently from Atlantic populations as a consequence of isolation and bottlenecks, as well as selection on adaptive traits^80^. Nonetheless, partial genetic separation might have occurred before formation of the Baltic Sea^61^. By analyzing 3 allozyme loci Moth-Poulsen^81^ indicated a gradual transition/cline between North Sea and Baltic Sea cod, i.e. a potential intraspecific hybrid zone, this was later confirmed by the analysis of nine highly variable microsatellite loci^29^. Strong differentiation between the east and west Baltic stocks was indicated by SNPs^61,62,69,82^. The analysis of SNPs presented here showed a difference between west samples including North Sea, Kattegat and west Baltic Sea, and Baltic Proper (south-eastern) samples. This divergence was represented by a clear split between the analyzed 9 populations and clustering of samples from the west Baltic together with samples from North Sea, *F*_ST_ values reduced 10-fold and showed a lack of haplotypes shared with samples from the East Baltic Sea. In this study, the isolation-by-distance (IBD) between samples tested by Mantel test for all 8076 SNP loci was significant and correlation between genetic diversity and geographic distance and bottom salinity were detected. The PCoA results suggested that the main differentiating factor could be explained by variable salinity represented by the 1^st^ axis what was further supported by results from outlier loci distribution and presence of different LDs associated with environmental factors. For outlier loci calculated for all 9 populations rapid change of maximum salinity level is the best explanation for the clear separation of groups from the North Sea and the Baltic Sea. Differences in salinity tolerance and subsequent low fitness of transplanted cod from the Baltic Sea and the Skagerrak/Kattegat^83^ and eastern (Gdańsk) and western (Kiel Bight) Baltic may be the result of genetically based adaptive differences between populations^52^, which potentially explain transcriptomic differences of *G. morhua* from the Baltic Sea that have also been observed^84^. Johannesson and André^80^ assumed that the cause of lost diversity of Atlantic cod was an efficient barrier to gene flow, which has evolved as a consequence of divergent selection on reproductive traits, such as egg buoyancy, sperm motility^73,85^ and different time of spawning season. Local adaptation of these traits can be manifested by selection evidence related with presence of outlier loci and their relations can be detected across genome by analysis of linkage disequilibrium^4,86^. The observed patterns of detected LD distribution in western dataset are congruent with earlier studies which indicated the presence of large LD region located on LG2 in cod from North Atlantic^68,87^. It was suggested that they are associated with salinity and oxygen level at spawning depth^68^. It is important that we did not observe significant outlier loci from LG2 in Baltic dataset. Significant LDs for Baltic samples were located on LG12 and LG13 for analysis with Bornholm cod and only on LG16 for easternmost cod. In first case, observed LDs concerned loci ss1712298167 described as associated with surface temperature and loci ss1712298916 located on important scaffold 08672 associated with many environmental correlation including surface and bottom salinity, oxygenation and temperature^68^. LDs detected in easternmost dataset also concerned outliers associated with bottom salinity (ss1712298846 and ss1712298845, scaffold 08549)^68^ but they were located on different LG and this may be related to the existence of adaptation to lowering salinity in the Baltic Sea from west to east. Furthermore, these loci were not indicated as outliers in western dataset and Bornholm cod.

The cod stocks in the North Sea and Kattegat were described as an indicator of the condition of Atlantic cod populations^88,89^. In present study with a large number of SNP loci, the Kattegat sample was closely related to the Scottish cod suggesting a high share of the North Sea cod. A low pairwise difference between North Sea and Kattegat samples has also been reported with microsatellites^29^, and may be explained by the significant transportation of cod larvae from the North Sea stocks into Kattegat^90^. High connectivity with offshore populations in Scandinavian fjords has been characterized recently using a large number of SNPs^38^. In the current study a sample of cod collected from the North Sea (Egersund fjord, Norway; EGR) was found to be slightly statistically different from the Kattegat and West Baltic, which coincides with presumably different local spawning areas in the western Baltic (Kattegat, Sound, Kiel and Mecklenburg Bays). However, analysis of the outlier loci for the group of samples from North Sea, Kattegat and western Baltic showed inconsistency between geographic origin and genetic distance of samples. The samples from the Egersund fjord (EGR) differed both from the Moray Firth and Kattegat samples. Genetic characteristics of the EGR samples could be potentially occurred due to a relatively closed coastal population breeding locally.

Low genetic distance among samples from EGR, Øresund (ORE), and Schlei fjord (SCH) suggests closer relationships with cod living under similar environmental conditions characterized by periodically reduced salinity^91,92^. Samples from EGR and SCH shared the same haplotypes (Table 4), which resulted in self-assignment of a significant percentage of individuals (40.74%) from Egersund fjord to the SCH sample. Despite high connectivity between populations caused by migration, cod populations could be characterized by adaptive differences influencing genetic differentiation. The association of genomic signatures and ecotypic divergence was noted by Hemmer-Hansen *et al*.^37^ and results presented here seem to support this conclusion.

The most divergent sample in the Baltic Proper is Bornholm (BOR), which was collected in July, after the spawning season, in order to avoid the migrants from other sub-locations. This sample, when tested with outliers, showed a relatively high value of *F*_ST_, distinguishing this population from other Baltic samples (*F*_ST_ = 0.06–0.09, P < 0.000). The genetic structure analysis showed that eastern Baltic stock samples formed four close clusters. This supports the assumption that the analyzed samples included individuals representing local populations, not the migrants. Genetic variability in the Baltic Proper samples was much lower than among western samples including also the west part of the Baltic Sea. The values of *F*_ST_ were lower suggesting that some specimens from each sample share the most functional spawning area in the Bornholm Deep. This is reflected in the results of the assignment tests where no clean baselines were observed. This is also indicator of high gene flow and significant level of mixing within the stocks. Additionally, low self-assignment of samples from Bornholm area with high share of easternmost stock is another argument that eastern Baltic cod occur in SD 24 on west cost of Bornholm what was also clearly demonstrated in recently published study by Hemmer-Hansen *et al*. 2019^61^.

Since the mid-1980s, successful spawning of eastern Baltic cod stock has been generally restricted to the Bornholm Basin^72,93,94^. The data here suggest that, thanks to the salt water inflows, spawning areas like Gdańsk Deep might have retained limited functionality in supporting divergence between local subpopulations^95,96^. The salinity factor seems to support more the divergence between west and east Baltic Sea than local divergence^57^. The potential spawning area is also determined by oxygen availability^54^. Genetically divergent but geographically close subpopulations have been identified, for instance, in Icelandic waters^97^, in the North Sea^88^, and along the Skagerrak coast^57,98,99^. For such differentiation to be preserved - even for small genetic differences - reproductive isolation is implied. In the Baltic Sea, local niches settled by cod are characterized by a unique set of environmental features like the diurnal and seasonal exchange of water masses^100^, vertical distribution of salinity and temperature^101^. These features and homing behaviour affect the genetic profile of the local subpopulation and maintain the distribution of genes/alleles responsible for local adaptations^22,38,52,69,84,89^^.^

Population genetic analyses facilitate detection of mixed stocks within the management units, and catch quotas are estimated on the assumption that a management unit includes only one stock. If more than one stock occurs in a management unit, the less abundant component becomes overfished and may collapse^28^. Studies of the relationship between population units and ICES subareas for North Sea Atlantic cod (*Gadus morhua* L.) have revealed that the genetically derived population units did not map accurately enough onto the existing cod management units^35^. One strategy for compensating for this situation is extending the spawning closure areas^72^. In Norway, along the coastal area, cod stocks were divided into two: north and south of 62^o^ latitude. Finding genetic structures along the Norwegian coast line by sampling 55 locations and analysing microsatellites^28^ and the pantophysin (*Pan* I) locus^102^ provided strong evidence to support possible revision of cod management strategy. The resilience of cod populations in the Kattegat may also be different when considered on smaller spatial scales than those delineated by traditional stock management boundaries^99^. Cod populations in the Baltic and Danish Straits have been managed for fishery purposes as 3 stocks differing in morphometric and genetic structures: Kattegat (subdivision 21), western (22–24) and eastern (25–32) Baltic^60,103–105^. Similarly to other studies, here we observed variation at the SNP loci between both Baltic stocks. Genetic differentiation between samples from the western and eastern Baltic stocks has been indicated as a tool for separation of western and eastern Baltic cod in mixed stock occupied SD24^61^. In addition, using outlier SNPs, this study was able to demonstrate genetic differences among populations from subdivisions. Genetic differences revealed between GDN and LIT (subdivision 3d 26) samples of cod collected from the eastern Baltic stock were supported by statistical analyses. Despite strong mixing, possible hindrances in connectivity between the Gdańsk Deep and Gotland Basin can be considered as explanation for the observed spatial differentiation of the eastern Baltic cod stock.

## Conclusions

This study demonstrated that genotyping with Norwegian cod SNP array constructed in CIGENE enables detection of genetic differentiation at a fine and local geographic scale in marine pelagic cod populations in the Baltic and adjacent waters. The sensitivity of the array towards identification of cod stocks can be enhanced by putting larger number of SNPs on the chip, including those polymorphic in the Baltic cod. Outlier SNPs are more informative markers in finding differences between Baltic cod populations in comparison with neutral SNPs. Here, with outlier SNPs, differentiation was identified between cod populations from subdivisions of existing management units in the Baltic. A tentative discrepancy between Lithuanian (LIT) and Polish (GDN) cod samples within one subdivision was also observed and can be related to possible isolation by environmental barriers between spawning areas in the Gdansk Deep and Gotland Basin. It is recommended to carry out further survey of Baltic cod populations using advanced genetic techniques on a larger number of specimens including larvae in order to further document the observed genetic pattern in the eastern Baltic cod population. Changes in time in genetic composition of Baltic cod stocks may be anticipated after periodic restrictions on fishing activities.

## Materials and Methods

### Sampling, DNA isolation and genotyping

**Figure 10:**
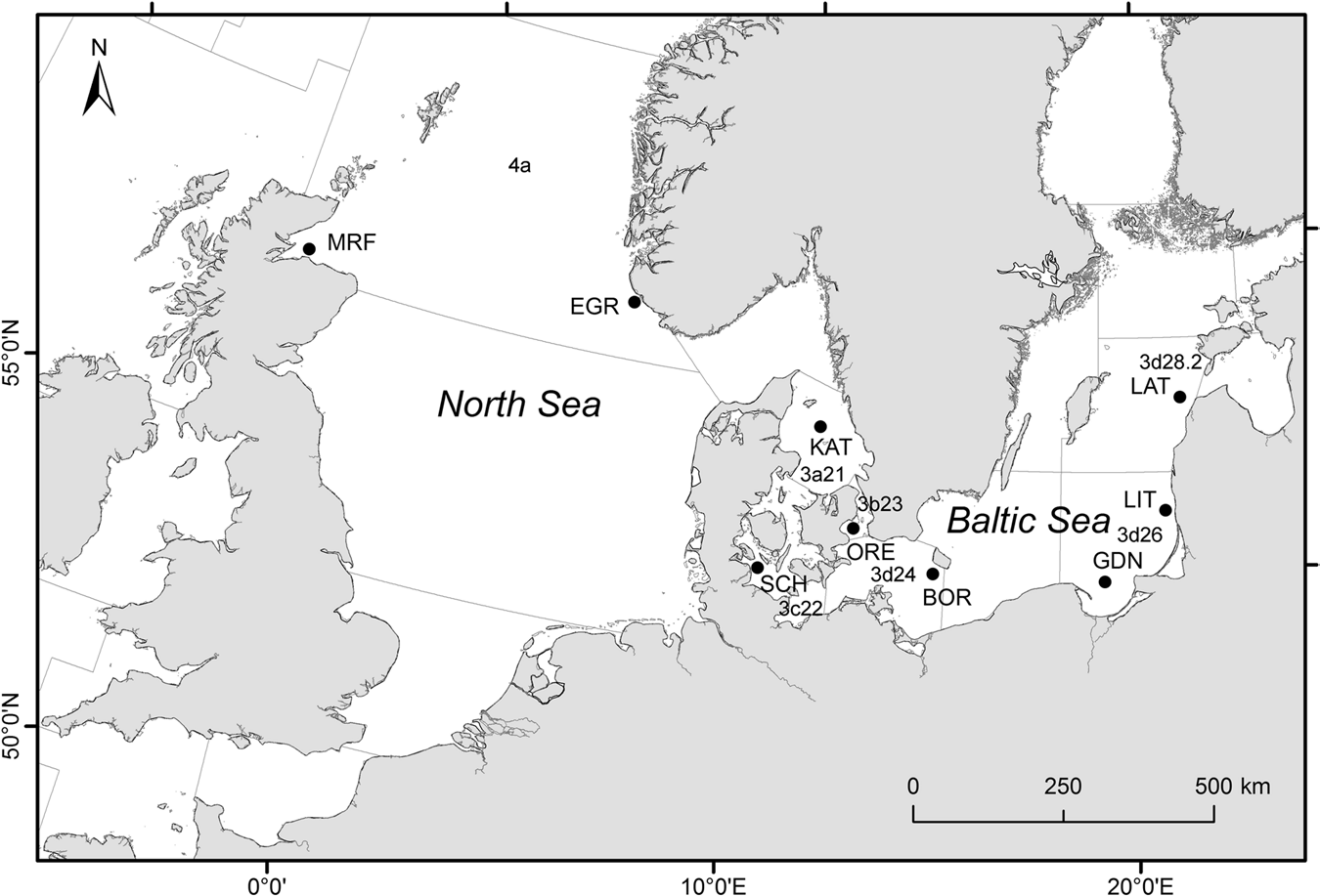
Map showing sampling sites and ICES subdivisions. Samples locations and codes are detailed in Table 5. Thin lines show borders between ICES subdivisions.

A total of 240 cod individuals from 9 locations at 7 ICES (*International Council for the Exploration of the Sea)* subdivisions along a transect across the Baltic Sea, Kattegat and North Sea (Fig. 10, Table 5) were collected between October 2012 - August 2013. Fin clips were stored in 70% ethanol at −70 °C. Genomic DNA was isolated using the Qiagen DNeasy 96 blood and tissue kit according the manufacturer’s instructions and stored at −20 °C. The concentration of DNA was determined by UV-vis spectroscopy using an Epoch Microplate Spectrophotometer (BioTek Instruments, Inc., Winooski, USA). After normalization, samples were genotyped on a custom *Gadus mohua* SNP-array (Illumina, USA) containing 10,923 SNP assays, and developed by a Norwegian consortium composed of four research organisations: Norwegian University of Life Sciences (NMBU), University of Oslo (UiO), NOFIMA AS, and the Institute for Marine Research (IMR)^38,68,69^. Samples were processed according manufacturers instructions and genotypes obtained from Genome Studio (V2011.1). After filtering to remove poorly clustering SNPs (failing assays, multisite variants), a total of 8221 diploid SNPs remained. This data set was further trimmed to remove: SNPs with relatively a high missing data level (over 20%; n = 15), monomorphic SNPs (n = 32), and SNPs with minor allele frequencies (MAF) < 0.01 (n = 98). The final data set included genotypes from 8076 loci.

**Table 5:** Numbers of examined cod specimens, sampling sites and ICES subdivisions in the Baltic Sea and North Sea.

Population	n	Sampling site	Date	Subdivision	Sea
LAT	30	NW Saaremaa, Latvia	2013-07	3d 28.2	east Baltic Sea
LIT	30	Coast of Lithuania	2013-02	3d 26	east Baltic Sea
GDN	24	Bay of Gdańsk, Poland	2012-11	3d 26	east Baltic Sea
BOR	21	Bornholm, Denmark	2012-07	3d 24	east Baltic Sea
SCH	30	Schlei, Belt Sea, Germany	2012-10	3c 22	west Baltic Sea
ORE	21	Øresund, Denmark	2013-07	3b 23	west Baltic Sea
KAT	23	Kattegat, Denmark	2013-07	3a 21	transition area
EGR	27	Egersund, Norway	2013-03	4a	North Sea
MRF	34	Moray Firth, Scotland		4a	North Sea

All methods complied with EC Directive 2010/63/EU for animal experiments and were approved by the Local Ethics Committee on Animal Experimentation at Gdansk Medical University (decision no. 60/2012).

### Statistical analysis

Allele frequencies and MAFs in each sample were calculated from spreadsheet data using Arlequin v. 3.5.1.3^106^. Genetic structure was analyzed using the program STRUCTURE v2.3.4^107^ which assigns individual genotypes to a specified number of groups, K, based on membership coefficients estimated from the genotype data. The analysis for 9 cod population samples was conducted from K = 1 to 12 using a burn-in period of 100,000 steps followed by 200,000 MCMC (Monte Carlo Markov Chain) replicates with 5 iterations, assuming an admixture model. The most probable number of clusters was defined by calculating the ΔK value^108^ determined by Structure Harvester^109^. Clumpp v.1.1.1^110^ was applied to average cluster membership using the Large K Greedy algorithm. Output from Clumpp was visualized in Distruct v.1.1^111^.

Arlequin v. 3.5.1.3 was used to perform an Analysis of Molecular Variance (AMOVA) with number of permutations = 90,000. Variance among the cod populations was detected by STRUCTURE, among samples, among individuals within populations and within individuals. The differentiation was tested amongst pairwise fixation index *F*_ST_ estimates and inbreeding coefficient *F*_IS_ estimates. The number of polymorphic loci and genetic diversity was calculated by measuring observed and expected heterozygosity (H_o_ and H_e_) with p < 0.05 and with exact test using a Markov chain with chain length =1,000,000 and dememorization steps =200,000. To adjust P value for each pair in multiple tests, Bonferroni corrections were included. GenAlex 6.502 was applied to perform a principal coordinates analysis (PCoA)^112,113^. Assignment tests were conducted using GeneClass^114^ with the allele frequency-based method. This enabled the identification of potential migrants or their descendants^115^. Relationships among 9 cod populations were examined using Poptree2^116^ with neighbor-joining (NJ) method based on *F*_ST_ distance with sample size correction^117^ and the number of bootstrap replications at 1000. The Mantel test based on dissimilarity matrices^118^ was applied to investigate the significance of relationships between genetic distance, geographic distance and bottom salinity with 999 permutations used to test the statistical significance of the values in GenAlex 6.502. Results were cross validated in Arlequin 3.5.1.3. Bottom salinity values were obtained from models GETM^119^, BALANCE^120^ and INSPIRE^121^. The hierarchical island model, implemented in Arlequin, was used to detect outlier loci. Loci as candidates under selection exhibited *F*_ST_ values out of the 99% quantile, based on coalescent simulations (50,000). Outlier loci were calculated with 50,000 simulations and number of demes at 100. Outlier loci were segregated and those with *F*_ST_ ≤ 0 or with *F*_ST_ > 0.01 were excluded. Separate structure investigation of outlier loci for west and east Baltic populations were carried out with increased burning (2,000,000) and MCMC (4,000,000). GenAlex was applied to perform a principal coordinates analysis (PCoA). Linkage disequilibrium (LD) was estimated for outlier loci by calculating the square value of correlation coefficient (r^2^) between pairs of markers^122^ using the TASSEL 5.2.58 software^123^. A threshold of r^2^ > 0.8 was considered to indicate LD. The level of LD was estimated for the entire panel and for the specific subgroups identified with STRUCTURE v2.3.4. Within these subgroups, LD was calculated considering only the detected panel of candidate outlier loci. The p-values for each r^2^ estimate were obtained with a two-tailed Fisher’s exact probability test and a threshold of p < 0.0001 was considered as significant. LD visualization was done by heat maps based on P values for pairwise r^2^ estimates to assess the overall view of LD patterns and evaluate LD blocks in various chromosomes at specific map locations. Additionally the distribution and clustering of detected outlier loci on linkage groups (LG) were indicated by Manhattan plots constructed for same subsets as for LD analysis using the R package “qqman”^124^. Homology searching was done through BLAST search of the available flanking sequences^125^ for each detected outlier loci on the NCBI and Ensembl public databases^126^. Functions of annotated outlier loci were determined using UniProt database. These analyses were done for three selected ouliers datasets specific for all Baltic, west Baltic and east Baltic (Supplementary Information Figure S1, Tables S1–S5).

## Supplementary information

Supplementary information.
