# Supplementary material for: SNP genotyping reveals substructuring in weakly differentiated populations of Atlantic cod (Gadus morhua) from diverse environments in the Baltic Sea
Source: Sci Rep. 2020 Jun 16;10:9738. doi: 10.1038/s41598-020-66518-4 (PMC7298039; doi:10.1038/s41598-020-66518-4)
Supplement: Supplementary file 1 — Supplementary information. [file 41598_2020_66518_MOESM1_ESM.pdf]

Supplementary information

SNP genotyping reveals substructuring in weakly differentiated populations of Atlantic cod (*Gadus morhua*) from diverse environments in the Baltic Sea

Roman Wenne, Rafał Bernaś, Agnieszka Kijewska, Anita Poćwierz-Kotus, Jakob Strand, Christoph Petereit, Kęstas Plauška, Ivo Sics, Mariann Árnyasi, Matthew P. Kent

Scientific Reports. https://doi.org/10.1038/s41598-020-66518-4

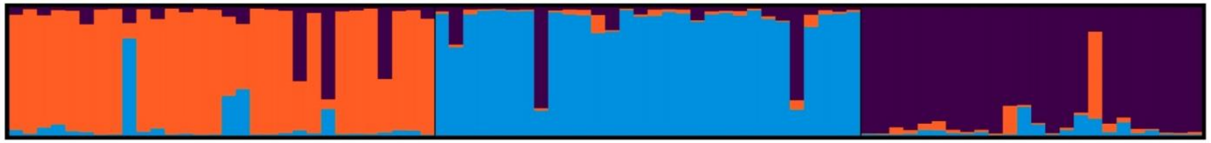

Figure S1. Graph represents the STRUCTURE results for the east Baltic dataset, based on 76 outlier loci and best K=3.

Table S1. List of 588 outlier SNPs used for analysis of all 9 cod samples.

SNP ID	ID	He	Obs. Fst	P-value
Gdist:475319_4922	3949	0.53358262	0.5892662	0.0000000000
NS:475319_4922	7529	0.53276521	0.58728104	0.0000000000
CAN:rs119056211	116	0.52689783	0.58425319	0.0000000000
NS:475319_4800	7528	0.53018893	0.56826752	0.0000000000
Gdist:285988_206	2166	0.50230948	0.54101946	0.0000000000
Gdist:403643_614	3484	0.50227845	0.49975545	0.0000000000
NS:88889_5530	8013	0.52953428	0.49782384	0.0000000000
Gdist:361153_730	3074	0.49252723	0.49088333	0.0000000000
GENE:18647_1548	6006	0.47452074	0.48531338	0.0000000000
Gdist:427747_995	3682	0.49800019	0.47816157	0.0000000000
Gdist:137108_1314	805	0.49662352	0.4720223	0.0000000000
Gdist:304686_764	2429	0.49662352	0.4720223	0.0000000000
Gdist:314227_2182	2519	0.49662352	0.4720223	0.0000000000
Gdist:245846_4065	1782	0.48899715	0.47103459	0.0000000000
Gdist:137184_860	807	0.4953752	0.46764949	0.0000000000
Gdist:192507_8811	1364	0.40969934	0.48533625	0.0000000000
NS:114636_2289	6718	0.52595149	0.43921002	0.0000000000
Gdist:391605_510	3366	0.52540926	0.43836236	0.0000000000

Gdist:39217_3257	3371	0.48757596	0.43567934	0.0000000000
CAN:rs119055764	47	0.48608002	0.43339426	0.0000000000
NS:95242_9678	8054	0.52666731	0.42717721	0.0000000000
Gdist:18651_2893	1287	0.46923215	0.42643218	0.0000000000
Gdist:397905_605	3417	0.50283092	0.42075694	0.0000000000
Gdist:106874_1573	419	0.50789208	0.41828077	0.0000000000
Gdist:742735_1353	5337	0.49074436	0.40871545	0.0000000000
Gdist:03217_1827	219	0.49806076	0.40578405	0.0000000000
Gdist:26927_508	2013	0.49806076	0.40578405	0.0000000000
Gdist:304748_348	2430	0.47733433	0.4067853	0.0000000000
Gdist:446823_143	3796	0.49895805	0.40298278	0.0000000000
Gdist:686846_6471	5099	0.49895805	0.40298278	0.0000000000
Gdist:176334_745	1196	0.45497384	0.40742927	0.0000000000
Gdist:205609_1348	1488	0.47800042	0.4003084	0.0000000000
NS:95242_6677	8053	0.51740777	0.39413984	0.0000000000
GENE:43253_2313	6205	0.52351743	0.39195347	0.0000000000
LD:184290_3102	6412	0.49893083	0.39245113	0.0000000000
Gdist:709175_544	5160	0.49600872	0.39211393	0.0000000000
GENE:391588_666	6167	0.52374178	0.38632475	0.0000000000
Gdist:233797_682	1694	0.49039282	0.38592175	0.0000000000
Gdist:137128_92	806	0.46666886	0.387149	0.0000000000
Gdist:370216_1271	3183	0.47343359	0.38517188	0.0000000000
Gdist:39236_1384	3377	0.44389746	0.38634253	0.0000000000
Gdist:135653_195	796	0.48414846	0.38028747	0.0000000000
NS:82343_3580	7981	0.5129157	0.37689671	0.0000000000
Gdist:356005_2291	2998	0.48534082	0.37710755	0.0000000000
Gdist:708727_3626	5157	0.52241021	0.36763506	0.0000000000
Gdist:282589_514	2133	0.40847906	0.40495929	0.0000000000
NS:22626_277	6967	0.43773662	0.3623372	0.0000000000
NS:475319_4758	7527	0.45594226	0.35529129	0.0000000000
LD:184288_1162	6411	0.48789427	0.34498867	0.0000000000

Gdist:157358_1355	1035	0.51939307	0.33931294	0.0000000000
Gdist:523966_606	4150	0.51799601	0.33905518	0.0000000000
GENE:523966_606	6244	0.51799601	0.33905518	0.0000000000
Gdist:358378_583	3026	0.45072381	0.34104393	0.0000000000
Gdist:318065_3383	2557	0.51218918	0.33031556	0.0000000000
Gdist:104640_1007	384	0.50457312	0.32942665	0.0000000000
Gdist:432966_985	3707	0.44885425	0.3326173	0.0000000000
Gdist:192904_2354	1376	0.50493519	0.32642354	0.0000000000
NS:58607_7078	7709	0.51538098	0.32526167	0.0000000000
Gdist:248573_1015	1803	0.49634162	0.32189213	0.0000000000
Gdist:53268_1290	4214	0.50849161	0.31998978	0.0000000000
NS:95239_3520	8052	0.51558638	0.31802337	0.0000000000
Gdist:708725_828	5156	0.51590793	0.31733498	0.0000000000
Gdist:30802_2497	2469	0.51094754	0.31431498	0.0000000000
Gdist:549050_3966	4318	0.51193063	0.31331773	0.0000000000
NS:51047_663	7557	0.48310675	0.31239476	0.0000000000
Gdist:330604_748	2705	0.51045437	0.31202192	0.0000000000
Gdist:794632_2777	5503	0.48670127	0.30781845	0.0000000000
Gdist:248559_173	1802	0.49823329	0.30419737	0.0000000000
Gdist:740626_1048	5324	0.51158866	0.30297508	0.0000000000
GENE:381177_2838	6160	0.48751607	0.30126289	0.0000000000
Gdist:358038_3346	3020	0.50961067	0.30019073	0.0000000000
GENE:282564_689	6062	0.43841297	0.30385898	0.0000000000
Gdist:220964_1437	1616	0.45900021	0.30067565	0.0000000000
Gdist:90160_4786	5729	0.48797318	0.29323334	0.0000000000
Gdist:549096_3487	4320	0.51799936	0.29259336	0.0000000000
NS:16158_4852	6836	0.50837298	0.2923152	0.0000000000
Gdist:00469_3262	187	0.51650323	0.28744195	0.0000000000
Gdist:304677_873	2428	0.51577314	0.28744261	0.0000000000
Gdist:68227_10928	5079	0.50355541	0.28616996	0.0000000000
GENE:322070_643	6110	0.51476793	0.28525086	0.0000000000

GENE:248219_2119	6030	0.46286711	0.2887386	0.0000000000
NS:248219_2119	6992	0.46286711	0.2887386	0.0000000000
Gdist:709168_1365	5159	0.51033909	0.28399286	0.0000000000
Gdist:87073_1942	5665	0.41775951	0.32626719	0.0000000000
Gdist:38911_3951	3332	0.51771065	0.28113056	0.0000000000
Gdist:502675_1253	4053	0.35836957	0.32467736	0.0000000000
NS:114393_697	6712	0.49083522	0.27467463	0.0000000000
GENE:282564_252	6060	0.51539264	0.27350704	0.0000000000
Gdist:41498_3248	3602	0.39649278	0.3179746	0.0000000000
NS:41498_2884	7429	0.39270683	0.31739737	0.0000000000
GENE:310389_1240	6091	0.51185053	0.27170995	0.0000000000
Gdist:276623_357	2076	0.47377811	0.27317053	0.0000000000
Gdist:643301_1935	4781	0.51172254	0.26896301	0.0000000000
GENE:398988_3229	6170	0.42530515	0.31083739	0.0000000000
Gdist:665768_709	4927	0.51335571	0.26717505	0.0000000000
NS:207040_1618	6939	0.51115968	0.26714689	0.0000000000
Gdist:137815_1452	820	0.34642155	0.30071954	0.0000000000
Gdist:300980_303	2360	0.35497742	0.30767092	0.0000000000
NS:273285_4421	7045	0.46343845	0.26593637	0.0000000000
Gdist:315835_1288	2533	0.4918219	0.26088545	0.0000000000
NS:364222_4453	7286	0.51498189	0.25941085	0.0000000000
Gdist:342952_3812	2861	0.51273761	0.25718077	0.0000000000
GENE:398989_637	6173	0.51535397	0.2564985	0.0000000000
Gdist:10872_2128	451	0.42860527	0.29895599	0.0000000000
Gdist:686813_660	5098	0.43440267	0.26110565	0.0000000000
LD:52208_643	6476	0.50474425	0.25550455	0.0000000000
Gdist:301945_295	2389	0.45879195	0.26003151	0.0000000000
Gdist:114631_3216	544	0.51299405	0.25441645	0.0000000000
NS:502686_4025	7553	0.34818354	0.28340288	0.0000000000
NS:07352_447	6650	0.4863117	0.24927275	0.0000000000
Gdist:263186_2996	1930	0.51054861	0.2484469	0.0000000000

Gdist:08560_1753	298	0.49783842	0.2470138	0.0000000000
Gdist:205817_112	1493	0.5143353	0.24545755	0.0000000000
GENE:381178_395	6162	0.46644171	0.24886257	0.0000000000
GENE:322070_2353	6109	0.42957841	0.28674924	0.0000000000
Gdist:312208_1203	2505	0.36858047	0.28845748	0.0000000000
Gdist:539182_884	4252	0.4242333	0.28706341	0.0000000000
Gdist:188071_251	1300	0.43241212	0.27603501	0.0000000000
NS:220985_2352	6964	0.48479863	0.23813302	0.0000000000
Gdist:431818_520	3698	0.40752572	0.28072655	0.0000000000
GENE:31134_1596	6102	0.51319327	0.23505394	0.0000000000
NS:33940_917	7215	0.4745514	0.23657727	0.0000000000
Gdist:33553_168	2763	0.51396574	0.2298974	0.0000000000
Gdist:86856_7607	5657	0.47262803	0.23275176	0.0000000000
Gdist:38012_1968	3264	0.50249717	0.22944157	0.0000000000
Gdist:102281_1292	352	0.48645711	0.22926622	0.0000000000
GENE:88975_377	6373	0.50325346	0.22763349	0.0000000000
NS:171176_722	6854	0.49622847	0.22731376	0.0000000000
NS:89001_469	8016	0.39430564	0.27151006	0.0000000000
LD:475253_2413	6455	0.49197672	0.22655527	0.0000000000
NS:99024_2514	8088	0.48728326	0.22542377	0.0000000000
GENE:99025_478	6388	0.5014934	0.22233399	0.0000000000
Gdist:413346_208	3595	0.40475883	0.2670568	0.0000000000
NS:459903_6489	7479	0.46726795	0.22371927	0.0000000000
Gdist:230992_680	1682	0.4768323	0.22033702	0.0000000000
Gdist:78312_977	5471	0.47322685	0.2195288	0.0000000000
Gdist:286510_2172	2184	0.48685603	0.21745506	0.0000000000
Gdist:134217_3155	786	0.5090099	0.21680112	0.0000000000
NS:134217_3155	6776	0.5090099	0.21680112	0.0000000000
Gdist:549068_926	4319	0.40543787	0.26029957	0.0000000000
NS:549068_926	7646	0.40543787	0.26029957	0.0000000000
Gdist:68779_1970	5104	0.46793324	0.2176238	0.0000000000

Gdist:320502_666	2581	0.51213215	0.21353049	0.0000000000
Gdist:105729_2123	401	0.50222518	0.21188843	0.0000000000
Gdist:26962_684	2015	0.41513846	0.25518897	0.0000000001
Gdist:120274_1489	609	0.43288693	0.21629208	0.0000000001
Gdist:388205_336	3326	0.51097916	0.20973475	0.0000000001
GENE:310389_3050	6095	0.47538687	0.21133252	0.0000000001
Gdist:330857_886	2709	0.50141905	0.2089358	0.0000000001
NS:134217_3861	6777	0.50590819	0.20867606	0.0000000001
NS:44697_2357	7464	0.51288316	0.20770159	0.0000000002
Gdist:340939_1382	2821	0.51294134	0.20756539	0.0000000002
Gdist:411787_875	3576	0.51261968	0.2061496	0.0000000003
GENE:381177_757	6161	0.46297193	0.21080339	0.0000000004
Gdist:105141_5763	391	0.45772931	0.21124856	0.0000000004
Gdist:314246_882	2520	0.49994921	0.20640105	0.0000000004
QTL:33228_656	8133	0.49940439	0.20582579	0.0000000005
Gdist:626777_1487	4699	0.50361929	0.2054753	0.0000000006
GENE:112790_1055	5922	0.39907211	0.24904094	0.0000000010
NS:304743_77	7137	0.46098415	0.20826751	0.0000000011
NS:105141_4988	6678	0.45903387	0.20839817	0.0000000011
NS:105141_5846	6679	0.45903387	0.20839817	0.0000000011
NS:731543_2272	7914	0.48744564	0.20397614	0.0000000012
Gdist:146167_456	921	0.4492793	0.20848681	0.0000000014
Gdist:321645_1106	2606	0.45098089	0.20812651	0.0000000015
GENE:207080_2407	6018	0.4961095	0.2023316	0.0000000021
Gdist:161401_294	1070	0.44727846	0.207332	0.0000000023
MOEN:rs119054621	6574	0.50699627	0.20071719	0.0000000033
Gdist:53094_285	4199	0.48824268	0.20076984	0.0000000040
GENE:207080_1795	6017	0.50164295	0.20005548	0.0000000047
Gdist:292756_416	2274	0.49080689	0.19999988	0.0000000052
NS:549059_1451	7645	0.45590631	0.20421717	0.0000000060
NS:80057_951	7967	0.50721809	0.19871091	0.0000000069

Gdist:260532_678	1903	0.45744394	0.20353789	0.0000000074
NS:76890_2534	7948	0.48307076	0.19873225	0.0000000083
Gdist:302657_494	2407	0.50393737	0.19808851	0.0000000092
CAN:rs119056093	94	0.39609511	0.2417276	0.0000000156
NS:24381_5221	6988	0.48992625	0.19679723	0.0000000166
Gdist:67076_4155	4956	0.4862129	0.19666569	0.0000000175
Gdist:67854_1143	5061	0.46884265	0.19939791	0.0000000176
NS:541375_1745	7635	0.51244026	0.19456999	0.0000000251
CAN:rs119055638	27	0.50912084	0.19472252	0.0000000265
Gdist:466957_178	3901	0.51118751	0.19413716	0.0000000304
Gdist:665785_2111	4928	0.49204868	0.19388702	0.0000000447
Gdist:351847_481	2963	0.45642511	0.19800009	0.0000000518
NS:463408_3089	7502	0.37556788	0.23740243	0.0000000587
NS:99436_3573	8091	0.45473188	0.19759511	0.0000000616
Gdist:523199_230	4142	0.51071238	0.19170074	0.0000000690
Gdist:74669_3025	5346	0.42196978	0.23429418	0.0000000740
Gdist:109466_1390	457	0.48256575	0.19236258	0.0000000745
Gdist:123803_258	659	0.47260961	0.1938665	0.0000000769
Gdist:248183_104	1795	0.45806682	0.19531662	0.0000001178
Gdist:261280_675	1910	0.45984765	0.19507928	0.0000001200
NS:201514_2254	6922	0.42713378	0.23005464	0.0000001378
Gdist:392165_819	3370	0.49769229	0.18948166	0.0000001773
GENE:282564_675	6061	0.5091227	0.1882412	0.0000002138
Gdist:43273_1498	3703	0.50237696	0.1885527	0.0000002231
Gdist:567155_188	4428	0.43595024	0.19443247	0.0000002235
Gdist:487211_430	3994	0.44996787	0.19338237	0.0000002566
Gdist:205713_12614	1491	0.49461763	0.18733437	0.0000003478
GENE:282586_980	6065	0.45723436	0.19101518	0.0000004434
Gdist:67055_2224	4955	0.51282878	0.18437955	0.0000005929
Gdist:98400_631	5878	0.50776714	0.18464866	0.0000006363
Gdist:182076_294	1244	0.51116772	0.18352005	0.0000007979

MOEN:rs119054722	6605	0.4344384	0.18791817	0.0000014739
NS:58607_7183	7710	0.36062433	0.22280858	0.0000016656
QTL:202454_289	8117	0.50998905	0.18021803	0.0000020124
GENE:310389_1490	6093	0.36268614	0.22231405	0.0000022278
Gdist:298541_1190	2333	0.5031319	0.18032022	0.0000022489
Gdist:391072_1596	3354	0.46649675	0.18345213	0.0000024114
Gdist:88987_3768	5705	0.51031289	0.17942216	0.0000024515
Gdist:380694_2686	3267	0.48310602	0.18027602	0.0000025206
Gdist:338891_1662	2801	0.50117962	0.17933078	0.0000029728
Gdist:266583_1995	1988	0.46208153	0.18285299	0.0000034235
GENE:56152_192	6249	0.4519755	0.1836848	0.0000035119
Gdist:310388_3476	2486	0.47285709	0.17974332	0.0000039446
Gdist:99610_355	5898	0.35546658	0.21502021	0.0000043977
Gdist:115290_227	554	0.50929208	0.17677413	0.0000048783
Gdist:539164_1195	4251	0.50203078	0.17637179	0.0000060980
NS:38798_2572	7354	0.50203078	0.17637179	0.0000060980
Gdist:146766_110	937	0.38272667	0.22028633	0.0000065881
GENE:67414_1576	6304	0.43210133	0.19579675	0.0000068679
Gdist:105669_1439	400	0.44364032	0.18148452	0.0000069780
Gdist:31123_2080	2494	0.4640747	0.17934783	0.0000073068
Gdist:302155_1821	2399	0.5036263	0.17551206	0.0000073277
Gdist:646840_181	4799	0.50174865	0.17522301	0.0000080191
Gdist:231727_417	1687	0.51194704	0.17396383	0.0000088333
Gdist:671659_1670	4976	0.50141955	0.17477472	0.0000089278
Gdist:85740_4265	5630	0.49968144	0.17450954	0.0000096480
Gdist:40022_842	3437	0.46111194	0.17846929	0.0000099271
NS:739927_4294	7921	0.36531269	0.21602342	0.0000102173
Gdist:77150_3853	5434	0.37038255	0.21681541	0.0000107413
Gdist:38796_3102	3321	0.50167387	0.17386281	0.0000109584
Gdist:209724_581	1534	0.50966077	0.17283495	0.0000121090
GENE:310390_643	6098	0.36466802	0.21492008	0.0000122411

Gdist:67010_6591	4953	0.50847588	0.17272997	0.0000127257
Gdist:94949_1204	5796	0.44175342	0.17863966	0.0000136387
Gdist:57173_191	4452	0.38960118	0.21606241	0.0000162907
Gdist:97627_7466	5863	0.50646024	0.17017664	0.0000229043
Gdist:50909_975	4071	0.36007522	0.2080642	0.0000299409
Gdist:79349_296	5499	0.41176088	0.21017589	0.0000329890
Gdist:40045_371	3438	0.50279121	0.16852254	0.0000336343
Gdist:110463_7123	477	0.43758746	0.17419942	0.0000354158
Gdist:60766_572	4623	0.28447825	0.20038081	0.0000365739
Gdist:21801_1426	1597	0.4192254	0.20714377	0.0000369650
NS:641386_799	7789	0.44332039	0.1735631	0.0000379296
Gdist:88878_402	5700	0.5031096	0.16553202	0.0000595365
LD:184291_1653	6414	0.38467453	0.20711	0.0000724374
Gdist:91357_1067	5750	0.50619319	0.16418402	0.0000736162
NS:40058_1135	7386	0.37339946	0.20584414	0.0000743161
NS:40058_2066	7387	0.37339946	0.20584414	0.0000743161
Gdist:119155_601	594	0.5014337	0.16399082	0.0000797332
GENE:515320_530	6240	0.30739332	0.19809136	0.0000956375
Gdist:101935_151	350	0.50799451	0.16249027	0.0000967603
Gdist:335662_1591	2767	0.47229555	0.16249685	0.0001284174
Gdist:84253_3311	5599	0.39746899	0.20214753	0.0001358673
NS:84253_3311	7988	0.39746899	0.20214753	0.0001358673
GENE:310389_1439	6092	0.37498549	0.20008241	0.0001556004
NS:63296_440	7784	0.35235406	0.18971838	0.0001626305
NS:248212_1732	6991	0.39528436	0.20066947	0.0001639127
Gdist:144553_3361	891	0.50425796	0.15922273	0.0001730077
Gdist:00611_735	191	0.3887386	0.2003346	0.0001733162
NS:63296_957	7785	0.47952252	0.1595384	0.0001745660
Gdist:322005_1780	2609	0.45883786	0.16268192	0.0001899972
Gdist:58607_9510	4515	0.37104752	0.19712586	0.0001999425
MITO:872249_152	6525	0.49501936	0.15801165	0.0002150240

Gdist:141142_1900	859	0.41073414	0.19401345	0.0002315692
Gdist:108688_456	450	0.48407568	0.15734941	0.0002411593
NS:539227_7884	7634	0.49667583	0.15681248	0.0002559993
Gdist:282564_987	2132	0.28064524	0.18702635	0.0002611002
GENE:282564_987	6063	0.28064524	0.18702635	0.0002611002
Gdist:12041_1466	612	0.47301347	0.15764405	0.0002619809
NS:63296_2282	7783	0.49810278	0.15659233	0.0002635364
NS:812321_8464	7970	0.41841343	0.18527315	0.0002843015
Gdist:11427_2980	531	0.43720017	0.16141476	0.0002906509
MOEN:rs119054574	6559	0.24394811	0.19833047	0.0002912480
NS:145687_509	6801	0.49552189	0.15588596	0.0002919007
Gdist:33945_203	2810	0.42093036	0.17986679	0.0002989372
Gdist:102613_4814	358	0.48366625	0.15571023	0.0003047324
Gdist:50279_689	4054	0.50954674	0.15444491	0.0003319066
Gdist:43235_3538	3702	0.41233719	0.18721405	0.0003407743
Gdist:563322_1175	4414	0.39194229	0.19287069	0.0003411223
Gdist:403958_874	3488	0.36715379	0.18979937	0.0003472858
Gdist:113412_760	523	0.51030278	0.15403578	0.0003477128
NS:565486_4514	7682	0.50266661	0.15441393	0.0003522011
Gdist:146790_1008	938	0.4782314	0.15428354	0.0003713734
Gdist:231731_2185	1688	0.26260583	0.17481188	0.0003803500
NS:231731_2185	6979	0.26260583	0.17481188	0.0003803500
GENE:207080_2858	6019	0.49443539	0.15380305	0.0003864176
Gdist:65559_1836	4847	0.45019301	0.1577433	0.0003981004
Gdist:539200_3087	4253	0.49691212	0.15346732	0.0004043368
Gdist:511825_1130	4085	0.4504117	0.15737437	0.0004149594
GENE:381181_1340	6164	0.42244476	0.16767185	0.0004183473
Gdist:623954_195	4680	0.49377978	0.15314017	0.0004211399
GENE:671175_2957	6302	0.41486211	0.17929453	0.0004243101
QTL:113257_1798	8100	0.50551143	0.15279327	0.0004314666
Gdist:145856_370	911	0.40210458	0.18745869	0.0004351947

NS:286176_399	7070	0.48709213	0.15262532	0.0004549288
Gdist:656035_2236	4854	0.50799625	0.15208817	0.0004637022
MITO:872249_575	6531	0.49945625	0.15171323	0.0005040759
Gdist:130189_4847	752	0.48741259	0.15179728	0.0005042641
NS:81980_3526	7975	0.4642445	0.1539555	0.0005428821
Gdist:371445_412	3188	0.47618699	0.15052888	0.0006106725
Gdist:32014_126	2571	0.4855704	0.15005003	0.0006204118
GENE:381176_275	6158	0.39395954	0.1806149	0.0006264827
Gdist:138934_1799	833	0.50888284	0.14957004	0.0006296570
NS:397494_4214	7380	0.48523578	0.14988171	0.0006328509
Gdist:40665_4865	3518	0.50872797	0.14940635	0.0006430745
GENE:310390_586	6097	0.39334838	0.1796044	0.0006473634
NS:39188_4049	7367	0.43835788	0.15468589	0.0006599255
Gdist:266374_1659	1981	0.47677525	0.14907121	0.0007168096
MOEN:rs119054557	6554	0.44867177	0.15272342	0.0007183347
Gdist:116909_2292	560	0.37769398	0.17093741	0.0007280670
Gdist:183663_7088	1268	0.48207297	0.14873372	0.0007343915
Gdist:76478_1805	5418	0.49623567	0.14840827	0.0007437596
Gdist:357812_7565	3013	0.41110647	0.16299226	0.0007573857
CAN:rs119055858	65	0.4111643	0.16255801	0.0007692741
Gdist:15542_744	1015	0.34640417	0.17599392	0.0007798160
Gdist:413145_1336	3591	0.49392949	0.14780392	0.0007917853
NS:270695_1166	7043	0.42256735	0.15649082	0.0008545545
Gdist:264304_175	1952	0.44399865	0.15162778	0.0008611696
Gdist:171234_4494	1154	0.41326848	0.15904583	0.0008721522
Gdist:344799_323	2885	0.49220072	0.14684436	0.0008818945
Gdist:32020_784	2573	0.39871877	0.16072348	0.0008953378
NS:95150_7188	8050	0.46041882	0.14886257	0.0009894265
Gdist:144574_1946	892	0.25920276	0.16583176	0.0010717816
Gdist:629713_580	4717	0.47818664	0.14523767	0.0010737701
Gdist:444927_575	3783	0.44502483	0.14913813	0.0010790060

NS:289612_6560	7087	0.49602398	0.14485032	0.0010829928
Gdist:307232_244	2459	0.46894846	0.14615254	0.0011399005
Gdist:554008_669	4362	0.4197269	0.15338897	0.0011429903
NS:643338_145	7794	0.4777941	0.14451052	0.0011550510
GENE:361218_1850	6142	0.41733282	0.15298317	0.0012116040
Gdist:341196_1962	2833	0.35900713	0.16578271	0.0012990252
Gdist:132254_1074	765	0.50708977	0.1429418	0.0013076890
Gdist:761338_106	5407	0.37739059	0.1548255	0.0013130149
Gdist:389980_612	3337	0.41573571	0.15184735	0.0013256825
Gdist:671648_3492	4975	0.50741738	0.1427713	0.0013278834
Gdist:270696_5455	2034	0.42754266	0.14957474	0.0013375641
Gdist:423219_94	3634	0.33654135	0.17358938	0.0013402454
Gdist:304766_2307	2431	0.33495854	0.17387195	0.0013828908
Gdist:129863_476	747	0.50737541	0.1423242	0.0013865822
Gdist:107715_804	439	0.49670387	0.14209966	0.0014138840
Gdist:827195_1380	5579	0.46353525	0.14463893	0.0014184244
Gdist:289623_3550	2230	0.49590613	0.14188173	0.0014395303
Gdist:548422_1314	4308	0.44382473	0.14610889	0.0014467891
Gdist:302969_239	2413	0.48628476	0.14188704	0.0014485372
CAN:rs119055778	52	0.47681523	0.14212515	0.0014674490
NS:314233_3663	7156	0.48175144	0.14193366	0.0014743598
GENE:192495_179	6011	0.200576	0.18976499	0.0014770138
Gdist:192234_1805	1355	0.48860082	0.14157622	0.0014886096
NS:545508_991	7640	0.33412155	0.17286643	0.0015279378
Gdist:132469_197	771	0.50300649	0.14131661	0.0015327672
Gdist:463341_214	3879	0.50619021	0.1412945	0.0015329818
Gdist:296079_657	2286	0.49639501	0.1410368	0.0015566131
Gdist:479481_402	3974	0.41822783	0.14938012	0.0015594584
Gdist:567225_906	4430	0.45249533	0.14379997	0.0015797730
Gdist:412025_75	3578	0.38030704	0.15105239	0.0016080795
NS:88984_1704	8015	0.25943662	0.16040152	0.0016379200

Gdist:567832_1870	4437	0.45358867	0.1429909	0.0016841142
NS:11598_1473	6726	0.50690265	0.14009942	0.0017056987
NS:338868_5248	7212	0.49413507	0.13987307	0.0017138387
QTL:643279_562	8166	0.44815454	0.14313586	0.0017328612
Gdist:370548_3747	3185	0.44859124	0.14242823	0.0018230193
Gdist:101435_3661	339	0.50813754	0.13930869	0.0018230865
Gdist:353176_174	2977	0.41122514	0.14719398	0.0018266700
Gdist:321324_396	2598	0.43843557	0.14370682	0.0018724544
GENE:56153_207	6250	0.50920727	0.13880133	0.0018977768
Gdist:794638_1797	5504	0.45740921	0.1412491	0.0019409971
GENE:343024_8049	6132	0.37080702	0.15208795	0.0019728736
Gdist:459894_790	3846	0.50444575	0.13829127	0.0020010692
NS:372638_143	7313	0.43782674	0.14288283	0.0020108942
Gdist:146397_340	927	0.31088365	0.17160393	0.0020254776
Gdist:739720_791	5303	0.47555576	0.13854194	0.0020430029
Gdist:427773_853	3683	0.24053153	0.16396996	0.0020626016
GENE:312795_1552	6104	0.50486919	0.1375132	0.0021353255
Gdist:423524_915	3639	0.46978331	0.13874415	0.0021610269
Gdist:475183_2114	3945	0.48909156	0.13708641	0.0021872857
Gdist:629798_2588	4720	0.50749448	0.13712069	0.0021982461
NS:04595_708	6635	0.50809068	0.13666456	0.0022790760
GENE:56152_1279	6248	0.50906345	0.1363988	0.0023225145
MOEN:rs119054754	6610	0.50205791	0.13631148	0.0023442202
Gdist:146741_1285	936	0.48824828	0.13620935	0.0023477015
NS:146741_1285	6810	0.48824828	0.13620935	0.0023477015
Gdist:46096_3323	3861	0.50041216	0.13573361	0.0024496444
Gdist:10682_1486	418	0.50857979	0.13560476	0.0024781879
Gdist:15851_436	1045	0.50096825	0.13546761	0.0025033980
NS:731543_2032	7913	0.43656435	0.14010129	0.0025209873
Gdist:08490_1670	296	0.48013846	0.13567765	0.0025292400
Gdist:525157_567	4154	0.29514075	0.16495748	0.0025367990

GENE:692219_2329	6324	0.50829803	0.13524261	0.0025513010
Gdist:338406_192	2787	0.43562369	0.13957838	0.0026401414
Gdist:73856_512	5299	0.48972554	0.13467676	0.0026412165
Gdist:542823_1947	4278	0.47062687	0.13564583	0.0027174901
NS:92962_107	8037	0.23582689	0.15832125	0.0027987338
GENE:338853_772	6120	0.45489255	0.13530307	0.0029754395
NS:179520_2574	6870	0.38628831	0.14090261	0.0029895569
NS:179520_1294	6869	0.50949087	0.13304746	0.0030070263
Gdist:214768_431	1563	0.35391181	0.15333994	0.0030186175
NS:214768_431	6949	0.35391181	0.15333994	0.0030186175
Gdist:146466_451	930	0.50068291	0.13292589	0.0030311914
Gdist:523202_934	4143	0.46954559	0.13421978	0.0030520676
Gdist:53439_892	4225	0.49341847	0.13249277	0.0030791498
Gdist:549293_10855	4325	0.32159538	0.16506209	0.0031045714
Gdist:739952_4766	5315	0.48257048	0.1325579	0.0031740660
Gdist:115198_1281	553	0.42492315	0.13894706	0.0031906018
Gdist:24362_1000	1762	0.50761882	0.1321663	0.0032156241
Gdist:195279_265	1405	0.47721447	0.13236391	0.0032688256
Gdist:109445_1327	456	0.44762171	0.13429152	0.0033024040
NS:367236_7276	7297	0.23255996	0.15322822	0.0033320615
NS:674746_3356	7852	0.43874532	0.13593226	0.0033469096
Gdist:220972_1997	1617	0.40807531	0.13825627	0.0033569416
Gdist:134377_139	788	0.39540125	0.138059	0.0033615697
NS:43236_1415	7450	0.42388734	0.13820272	0.0033958325
CAN:rs119056096	95	0.50653085	0.131389	0.0034015827
NS:24380_7441	6987	0.50204885	0.13132019	0.0034063345
Gdist:739832_3654	5308	0.41931951	0.1383943	0.0034503739
NS:401454_3578	7390	0.33096022	0.1608236	0.0035309585
Gdist:115190_1218	552	0.42829781	0.1368275	0.0035692379
Gdist:49577_5430	4024	0.4672248	0.13206573	0.0035980571
GENE:183615_3430	6001	0.50818246	0.13049736	0.0036181397

LD:475273_1292	6464	0.4629319	0.1317985	0.0037484818
Gdist:106913_2173	420	0.30094683	0.15962531	0.0037506798
NS:137099_523	6780	0.40626047	0.13618657	0.0038336204
Gdist:304651_233	2427	0.35002422	0.15068024	0.0038992242
Gdist:101023_2109	325	0.25718943	0.14178176	0.0040113706
Gdist:73474_7746	5267	0.48601749	0.12892165	0.0040310384
NS:201684_3818	6925	0.48918251	0.12855081	0.0041112075
Gdist:427869_3081	3686	0.2383206	0.14636118	0.0041325538
Gdist:322086_381	2612	0.31006608	0.15919295	0.0041428175
Gdist:351468_1797	2954	0.50426511	0.12848006	0.0041555083
NS:459921_4342	7482	0.43483676	0.1329322	0.0042334440
Gdist:89516_1823	5717	0.48625978	0.12815272	0.0042481763
GENE:95224_3138	6385	0.50756022	0.1281194	0.0042507420
Gdist:630523_2206	4729	0.50724806	0.1279104	0.0043105213
Gdist:317570_316	2553	0.49202193	0.12715472	0.0044844237
Gdist:143742_85	881	0.42129759	0.1341746	0.0045528896
Gdist:361332_2828	3081	0.50566222	0.12705362	0.0045655312
Gdist:64916_1498	4807	0.47403484	0.12788001	0.0045675180
Gdist:583203_2493	4495	0.40826517	0.13359134	0.0046331338
Gdist:71394_2079	5169	0.4614548	0.12857697	0.0046335528
Gdist:82446_620	5575	0.4322666	0.13148338	0.0047943923
Gdist:29800_1945	2325	0.29064296	0.15143974	0.0047973604
Gdist:386936_835	3306	0.42392497	0.13280405	0.0048727667
GENE:43254_587	6207	0.40564176	0.13263266	0.0048738454
Gdist:283627_731	2142	0.50565204	0.12569768	0.0049822953
Gdist:538311_232	4241	0.4715918	0.12664753	0.0050053479
GENE:614866_553	6277	0.48653516	0.12559549	0.0050399376
NS:402418_6400	7394	0.30301809	0.15336085	0.0051458609
Gdist:238805_341	1734	0.37561009	0.13582538	0.0051485796
Gdist:202746_5500	1459	0.32383639	0.15483207	0.0051990967
QTL:125149_1904	8102	0.47526589	-0.018135674	0.0052053932

Gdist:739921_2010	5313	0.50614362	0.12487635	0.0052446225
NS:64647_5114	7797	0.37500654	0.13567916	0.0052794466
Gdist:348894_914	2931	0.27480042	0.14375261	0.0053693811
Gdist:198328_243	1417	0.49820264	0.12437254	0.0053878380
LD:411401_1118	6442	0.36113789	0.14043996	0.0054529220
Gdist:19397_3287	1394	0.36715995	0.13760407	0.0054575253
Gdist:548325_2365	4305	0.41100107	0.13097052	0.0055503468
Gdist:306034_249	2439	0.48033783	-0.017514683	0.0055689142
Gdist:626853_560	4702	0.41693335	0.13100896	0.0056307598
NS:40015_377	7384	0.50265509	0.12365807	0.0056650066
NS:402418_7998	7395	0.29729712	0.1487606	0.0056989714
Gdist:276572_532	2073	0.27489378	-0.017821436	0.0057554587
Gdist:248208_883	1796	0.30003395	0.14973531	0.0057635833
Gdist:141364_2559	864	0.39082424	0.1311194	0.0057869985
Gdist:56160_1160	4404	0.34833139	0.14421669	0.0058924457
LD:52206_4207	6474	0.47133764	0.12405295	0.0059100667
NS:565515_954	7683	0.49997739	-0.016042287	0.0059197374
Gdist:105748_1200	402	0.4462389	0.12489135	0.0060389872
NS:129362_255	6762	0.37306397	0.13421455	0.0060660263
Gdist:62139_296	4675	0.50154899	0.12246709	0.0060983371
MOEN:rs119054746	6608	0.39082895	-0.016738861	0.0061165285
Gdist:30866_2166	2476	0.46809154	0.12366493	0.0061296026
Gdist:210178_764	1546	0.49158951	-0.016242005	0.0061757557
NS:385816_1167	7349	0.44469788	0.12500634	0.0061760417
Gdist:471821_1149	3926	0.44953942	0.12387786	0.0061964123
Gdist:146512_541	932	0.50433001	0.12214797	0.0062259524
Gdist:141386_2513	865	0.50044126	0.12210547	0.0062374079
LD:184286_794	6410	0.33627055	0.14756469	0.0062612008
NS:107642_1498	6689	0.25651597	0.13200744	0.0063311493
Gdist:210027_7466	1540	0.47870915	-0.016685562	0.0063963407
Gdist:335642_427	2765	0.49950264	0.12144149	0.0064926369

Gdist:463439_186	3882	0.50114165	0.12143197	0.0065041694
Gdist:350168_1259	2943	0.43122959	-0.016148544	0.0065850854
Gdist:57225_547	4456	0.49956282	-0.015364195	0.0066092154
Gdist:95799_179	5816	0.50228333	0.12092076	0.0067105314
Gdist:39080_4386	3344	0.39180867	0.12869667	0.0067244479
GENE:202734_512	6014	0.2885912	0.14204651	0.0067628443
Gdist:538340_560	4242	0.30679093	0.14748561	0.0067803030
Gdist:644561_942	4795	0.49907807	-0.015215942	0.0067901106
Gdist:157788_1708	1039	0.49653752	-0.015336244	0.0068125858
Gdist:05088_594	245	0.49835262	-0.015226575	0.0068236009
NS:56864_18298	7692	0.47646674	-0.016184329	0.0069979919
Gdist:123937_925	661	0.49951525	-0.014973929	0.0070251388
Gdist:285501_1826	2163	0.50712304	0.11967903	0.0072066570
Gdist:629637_1807	4714	0.38225473	-0.015482217	0.0072804619
Gdist:202762_5167	1460	0.50605687	0.11941341	0.0073368383
Gdist:43584_4197	3733	0.47498873	-0.015908358	0.0073512061
GENE:119497_633	5923	0.45671996	-0.015876038	0.0073948935
Gdist:51091_2121	4077	0.45039348	-0.015679505	0.0074301298
Gdist:138884_272	831	0.50739784	0.11913915	0.0074423994
NS:114389_364	6711	0.35263493	0.13808194	0.0074855516
GENE:86498_247	6365	0.50199009	0.11911031	0.0074975492
Gdist:774889_1160	5444	0.49298254	-0.014909467	0.0074996613
Gdist:574768_666	4464	0.41642257	-0.015177435	0.0075298092
CAN:rs119056130	102	0.32753297	0.1460906	0.0075322441
Gdist:172032_399	1167	0.47123407	-0.015820408	0.0075597207
GENE:444129_2205	6211	0.49504579	-0.01468166	0.0076234750
Gdist:68768_2445	5103	0.40359039	0.12548756	0.0077097187
CAN:rs119055776	51	0.49551476	-0.014567674	0.0077264723
Gdist:119497_633	602	0.45706545	-0.015576901	0.0077426705
GENE:692217_2781	6322	0.4695364	0.11966005	0.0077990318
Gdist:59381_4686	4557	0.34648075	-0.015212219	0.0078489905

Gdist:118515_4597	587	0.43636862	-0.0150321	0.0078558156
NS:154935_683	6825	0.25550584	0.12783527	0.0078842658
Gdist:82562_1187	5576	0.49887775	-0.014195922	0.0079556387
Gdist:189646_356	1324	0.39557113	-0.014821666	0.0079876425
Gdist:335655_1240	2766	0.49971666	0.11795378	0.0080523877
Gdist:262230_7414	1920	0.45016543	0.11933617	0.0081269570
GENE:95224_2887	6384	0.50808185	0.11754299	0.0081867617
Gdist:113502_4264	529	0.346156	0.13889824	0.0081937601
NS:144600_1820	6800	0.2611725	0.12806378	0.0082105763
Gdist:298095_597	2329	0.49079744	-0.014382096	0.0082561750
Gdist:361278_3241	3080	0.42855327	0.12312066	0.0082605157
NS:549045_140	7644	0.38090651	0.12761964	0.0082763856
Gdist:90141_2148	5728	0.49845202	0.11743643	0.0083058339
Gdist:341032_908	2826	0.45450982	0.11882429	0.0083810385
Gdist:401542_1083	3457	0.44731567	0.11907069	0.0083854797
Gdist:366965_6675	3144	0.47532974	-0.014984682	0.0084218179
Gdist:254208_2166	1842	0.39811032	-0.014386098	0.0084364172
Gdist:205485_1725	1483	0.20611713	-0.015874206	0.0084639897
Gdist:552740_1950	4346	0.28310256	-0.014738604	0.0085263515
NS:552740_1950	7658	0.28310256	-0.014738604	0.0085263515
GENE:102276_260	5914	0.46199497	0.11837669	0.0085944240
NS:124260_901	6750	0.24296081	-0.015829276	0.0086381385
Gdist:539698_99	4256	0.48569401	0.11714755	0.0086596291
NS:740608_5723	7924	0.17982241	0.13756742	0.0086909492
Gdist:381174_2967	3269	0.3281245	0.14265709	0.0087050316
Gdist:51185_460	4086	0.33604297	-0.01450564	0.0087123452
NS:286725_1479	7078	0.44761854	0.11836388	0.0087212105
Gdist:301650_1516	2377	0.49253331	-0.01392802	0.0087229607
Gdist:33636_619	2776	0.27554202	-0.014995764	0.0087240069
Gdist:231755_782	1689	0.29584631	0.13702904	0.0087839660
NS:96373_5508	8068	0.43080379	-0.014183719	0.0088348267

Gdist:422914_951	3632	0.47201103	-0.014734884	0.0088560436
NS:56149_5701	7676	0.4226931	0.12270539	0.0088587247
NS:674746_3398	7853	0.4698676	0.11753695	0.0088640813
Gdist:49916_70	4038	0.50474932	0.11628972	0.0088745993
NS:88549_899	8010	0.49943703	-0.013437635	0.0088773944
QTL:113201_1555	8096	0.42200375	0.12270891	0.0089073870
Gdist:93696_2438	5780	0.39889726	-0.013937919	0.0089766445
Gdist:208782_2306	1527	0.36618818	0.12950932	0.0090682734
Gdist:264125_795	1946	0.43488674	-0.014003065	0.0091177099
NS:670952_2234	7837	0.34744808	0.13634981	0.0091202998
Gdist:01044_7773	194	0.39449958	0.12341531	0.0091461918
Gdist:157990_234	1043	0.30073813	-0.013866601	0.0091497149
CAN:rs119055771	49	0.28336151	-0.014196144	0.0092110567
NS:92360_3713	8033	0.3361921	0.1393243	0.0092770909
NS:263966_1557	7029	0.40133729	0.12240388	0.0092984348
Gdist:06344_452	264	0.38673957	-0.013781356	0.0093042895
Gdist:198299_2477	1416	0.29437357	-0.013773232	0.0093283293
NS:674746_3509	7854	0.46731311	0.11673074	0.0093300331
Gdist:206951_3729	1508	0.42812218	-0.013795895	0.0093367914
Gdist:511757_1396	4082	0.47172834	0.11649525	0.0093911640
Gdist:423541_1016	3640	0.50766944	0.11512742	0.0094474318
Gdist:479496_3696	3975	0.37226339	0.12727373	0.0094613096
NS:03532_2275	6632	0.35689055	0.1320449	0.0095617995
Gdist:384842_221	3290	0.49617777	-0.013097506	0.0095762560
GENE:482589_2280	6228	0.48934159	-0.013435631	0.0096069837
MOEN:rs119054761	6615	0.49997477	-0.012872451	0.0096139727
NS:53236_3235	7628	0.49997477	-0.012872451	0.0096139727
Gdist:302314_1670	2404	0.29216629	-0.013584269	0.0096394463
Gdist:313292_688	2507	0.30221464	-0.01350971	0.0096396081
NS:183645_4623	6883	0.50416112	0.11488639	0.0096665640
Gdist:266350_1227	1980	0.37669849	-0.01340002	0.0096950893

Gdist:27817_905	2085	0.35251988	-0.013660362	0.0096963294
Gdist:46720_1713	3905	0.39586662	-0.01340028	0.0097462380
Gdist:101006_10283	324	0.43801326	0.11818977	0.0097533769
Gdist:321015_856	2594	0.46209948	0.11625966	0.0097634722
Gdist:16010_3095	1065	0.50071381	0.11475548	0.0097814766
Gdist:55893_2153	4397	0.48518931	-0.013534936	0.0097907487
Gdist:365850_1117	3123	0.39760214	-0.013304695	0.0098286857
Gdist:499661_1689	4040	0.37819664	-0.013292761	0.0098406620
Gdist:324346_1449	2645	0.47719833	-0.013851334	0.0098423031
NS:64647_5678	7798	0.32105753	0.13977297	0.0098469076
Gdist:109590_226	459	0.12461271	-0.015827698	0.0099293511
Gdist:662180_502	4888	0.49881017	-0.01268645	0.0099680566

Table S2. List of outliers used for analysis of 175 western Baltic, Kattegatt and North Sea populations.

**SNP ID**	**ID**		**He**	**Obs. Fst**	**P-value**
Gdist:340939_1382	2821	2759	0.5316007000	0.3557609700	0.0000000000
Gdist:192507_8811	1364	1325	0.5330413200	0.3245499500	0.0000000000
MOEN:rs119054557	6554	6430	0.3638126700	0.1922594200	0.0000000000
Gdist:146167_456	921	886	0.5151456000	0.2051748000	0.0000000000
Gdist:545739_884	4287	4203	0.4713276800	0.1845900300	0.0000000000
CAN:rs119056211	116	97	0.3993176500	0.1677899900	0.0000000000
Gdist:285988_206	2166	2112	0.4822660500	0.1808414800	0.0000000000
CAN:rs119055764	47	38	0.1938451100	0.1972943400	0.0000000000
Gdist:137108_1314	805	770	0.1938451100	0.1972943400	0.0000000000
Gdist:137184_860	807	772	0.1938451100	0.1972943400	0.0000000000
Gdist:304686_764	2429	2370	0.1938451100	0.1972943400	0.0000000000

Gdist:304748_348	2430	2371	0.1938451100	0.1972943400	0.0000000000
Gdist:314227_2182	2519	2459	0.1938451100	0.1972943400	0.0000000000
Gdist:427747_995	3682	3607	0.1938451100	0.1972943400	0.0000000000
GENE:282564_689	6062	5948	0.1938451100	0.1972943400	0.0000000000
Gdist:301945_295	2389	2331	0.5205133200	0.1762250500	0.0000000000
Gdist:137128_92	806	771	0.1811857500	0.1856619000	0.0000000000
Gdist:157358_1355	1035	996	0.3891556300	0.1513777200	0.0000000001
Gdist:08560_1753	298	274	0.5058466100	0.1666277900	0.0000000001
GENE:322070_2353	6109	5995	0.1873415800	0.1836031400	0.0000000001
Gdist:320502_666	2581	2521	0.4725814900	0.1522772400	0.0000000082
Gdist:94561_5380	5794	5683	0.4471356500	0.1369175000	0.0000000178
NS:270695_1166	7043	6915	0.4951352100	0.1483156900	0.0000000698
Gdist:584831_1222	4511	4421	0.1547810200	0.1610962400	0.0000001238
CAN:rs119056093	94	75	0.1680443900	0.1585740200	0.0000001732
Gdist:549068_926	4319	4235	0.1680443900	0.1585740200	0.0000001732
NS:549068_926	7646	7508	0.1680443900	0.1585740200	0.0000001732
Gdist:266583_1995	1988	1936	0.5160881900	0.1408320900	0.0000006367
Gdist:304651_233	2427	2368	0.1922030600	0.1571417300	0.0000010622
Gdist:106964_248	421	393	0.5158192300	0.1388754300	0.0000010851
Gdist:146790_1008	938	903	0.5164251500	0.1387567300	0.0000011107
Gdist:270696_5455	2034	1982	0.4973139400	0.1378610300	0.0000014662
Gdist:257206_1563	1878	1828	0.4723622300	0.1326084600	0.0000021537
Gdist:397905_605	3417	3345	0.2447433100	0.1531772100	0.0000053920
Gdist:49577_5430	4024	3943	0.3816836600	0.1157380700	0.0000068501
Gdist:342952_3812	2861	2798	0.4582391100	0.1174979800	0.0000086170
NS:515296_610	7570	7434	0.4590167300	0.1155819800	0.0000135274
Gdist:70440_330	5148	5045	0.4942191800	0.1268347600	0.0000175924
Gdist:146397_340	927	892	0.4181475600	0.1135337400	0.0000176205
GENE:192495_179	6011	5898	0.3190278200	0.1176135200	0.0000200372
GENE:381178_395	6162	6047	0.5147508700	0.1260777500	0.0000205468

GENE:146766_1682	5958	5846	0.4311860300	0.1065778600	0.0000708749
Gdist:21801_1426	1597	1555	0.5029504200	0.1186041200	0.0000800132
Gdist:33963_954	2811	2750	0.5111998600	0.1180404100	0.0000851291
NS:95242_9678	8054	7909	0.3514753900	0.1047232900	0.0000915844
NS:92962_107	8037	7892	0.3464571300	0.1054185700	0.0001011850
Gdist:403643_614	3484	3412	0.1794332500	0.1283373000	0.0001016166
GENE:381177_2838	6160	6045	0.4999949300	0.1145615900	0.0001436506
Gdist:106874_1573	419	391	0.2600404900	0.1320656300	0.0001656097
GENE:282564_252	6060	5946	0.3976059600	0.1000685700	0.0001758811
Gdist:146766_110	937	902	0.4869607800	0.1105794800	0.0002073228
Gdist:161401_294	1070	1031	0.5113474700	0.1108316600	0.0002268017
Gdist:192904_2354	1376	1337	0.3141609200	0.1020813700	0.0002615667
Gdist:205713_12614	1491	1450	0.5084421600	0.1073594800	0.0003438394
Gdist:157373_192	1036	997	0.1278743700	0.1212178500	0.0004001288
Gdist:08949_2193	305	280	0.5101424500	0.1051703400	0.0004256144
Gdist:90656_702	5735	5624	0.3229613000	0.0978808500	0.0004263827
GENE:282564_675	6061	5947	0.4839297700	0.1031084200	0.0004524526
Gdist:77904_146	5464	5356	0.3929245400	0.0932208300	0.0004593783
Gdist:157811_346	1040	1001	0.4705118800	0.0974523500	0.0005029505
Gdist:304677_873	2428	2369	0.3920935400	0.0915974000	0.0005654853
Gdist:38131_1235	3274	3202	0.4458543800	0.0919523900	0.0005711877
Gdist:104640_1007	384	359	0.3089252000	0.0952920000	0.0005951601
Gdist:90160_4786	5729	5618	0.4989626300	0.1015792100	0.0006166765
Gdist:281573_2257	2126	2073	0.4600012100	0.0919528500	0.0006709044
GENE:282586_980	6065	5951	0.5108774300	0.0991482300	0.0007282382
Gdist:423219_94	3634	3560	0.1467159000	0.1134147100	0.0008367576
Gdist:68504_1675	5090	4988	0.5100610800	0.0969301900	0.0008780935
Gdist:367146_4180	3152	3081	0.1404629400	0.1125675400	0.0009420308
NS:417075_807	7431	7298	0.4705157700	0.0892590100	0.0011784459
NS:171176_722	6854	6730	0.4975977600	0.0937580800	0.0011815811
Gdist:403191_1850	3480	3408	0.4675410800	0.0877902400	0.0012671445

Gdist:101935_151	350	325	0.4845806200	0.0907051800	0.0013914600
Gdist:549050_3966	4318	4234	0.3478797300	0.0843151300	0.0014239138
NS:344801_9796	7228	7098	0.3930705100	0.0833469100	0.0014816822
Gdist:08949_1031	304	279	0.5013134900	0.0894940700	0.0016244632
Gdist:295385_2637	2285	2230	0.5015073800	0.0884228800	0.0017570913
NS:194155_5768	6912	6787	0.4835936000	0.0872062400	0.0018234048
Gdist:298554_452	2335	2280	0.4950768700	0.0870481600	0.0019614220
NS:15275_1590	6821	6697	0.1279986900	0.0981840000	0.0021627717
Gdist:73512_4050	5271	5164	0.2856343000	0.0879695400	0.0022120123
NS:41498_2884	7429	7296	0.5046806000	0.0850824800	0.0022134831
NS:95242_6677	8053	7908	0.3095855600	0.0815336700	0.0022653156
Gdist:261280_675	1910	1860	0.3040243400	0.0816208500	0.0022901953
NS:95239_3520	8052	7907	0.4466962300	0.0791253000	0.0023926048
NS:545508_991	7640	7502	0.1339420500	0.0956434600	0.0024335234
Gdist:565410_1074	4420	4334	0.2202472900	0.0999639000	0.0024843425
Gdist:16010_3095	1065	1026	0.4530239300	0.0788368000	0.0025000304
CAN:rs119055638	27	18	0.4780876400	0.0822162800	0.0025515655
Gdist:366268_887	3126	3055	0.5094327700	0.0819932100	0.0026811739
Gdist:94547_597	5793	5682	0.2192412400	0.0978354400	0.0026888155
Gdist:439820_858	3761	3684	0.3133160500	0.0791864800	0.0027013269
Gdist:50909_975	4071	3990	0.1347516000	0.0926389000	0.0027406691
GENE:357806_2806	6135	6020	0.3033942500	0.0794253400	0.0027975557
Gdist:312208_1203	2505	2445	0.0799936900	0.0886595100	0.0029291643
Gdist:674947_1631	5013	4911	0.4097750400	0.0772292500	0.0029746359
Gdist:754476_1303	5379	5271	0.4899919500	0.0810897100	0.0030212146
Gdist:01044_7773	194	170	0.2680096800	0.0905078100	0.0030731920
Gdist:77609_1845	5454	5346	0.3639940600	0.0756202200	0.0030744235
Gdist:264304_175	1952	1901	0.2996836600	0.0787475500	0.0031028169
NS:01039_6498	6626	6502	0.1653949000	0.0867399500	0.0031648639
Gdist:338980_3381	2802	2741	0.3879263100	0.0757923100	0.0032287828
Gdist:163633_843	1094	1055	0.4564852400	0.0762047400	0.0032716083

Gdist:269670_678	2016	1964	0.3987483700	0.0755970800	0.0032910355
NS:63296_957	7785	7642	0.5087144500	0.0793012600	0.0033173813
Gdist:286585_1454	2186	2132	0.3475816600	0.0757686100	0.0033488652
NS:812321_8464	7970	7826	0.4987469900	0.0795299700	0.0034256208
Gdist:427869_3081	3686	3611	0.3521236200	0.0752169200	0.0034483816
Gdist:91939_856	5762	5651	0.2532337000	0.0929439300	0.0036115722
Gdist:549096_3487	4320	4236	0.4054497800	0.0745926500	0.0036998808
Gdist:336114_567	2772	2711	0.3072272500	0.0750655100	0.0039619925
Gdist:41498_3248	3602	3528	0.5049702700	0.0773148800	0.0039695703
NS:33940_917	7215	7085	0.2879143800	0.0779010200	0.0043308327
Gdist:182076_294	1244	1205	0.4481197400	0.0722054100	0.0044675683
NS:207040_1618	6939	6813	0.4602573700	0.0723509500	0.0045863868
Gdist:360398_615	3055	2984	0.4019992500	0.0721416000	0.0046043862
Gdist:32882_5027	2689	2628	0.5087990500	0.0745144700	0.0048094838
Gdist:476911_2677	3956	3875	0.4575977900	0.0715516300	0.0048694241
NS:616490_772	7761	7618	0.4575759100	0.0709668500	0.0051055152
Gdist:100823_1792	316	291	0.4908222900	0.0741202900	0.0051291332
Gdist:273139_6138	2050	1998	0.4363836300	0.0717112500	0.0051313226
Gdist:334110_720	2742	2681	0.2584993500	0.0838800300	0.0051966285
Gdist:59628_4649	4577	4486	0.5036233900	0.0729726500	0.0055823119
Gdist:14270_129	878	843	0.5049126900	0.0728056900	0.0056232466
Gdist:282589_514	2133	2079	0.0515379100	0.0752637100	0.0056763786
Gdist:09836_3133	310	285	0.4050643200	0.0698630700	0.0057255326
Gdist:740626_1048	5324	5217	0.3476785900	0.0698473600	0.0057773604
Gdist:296897_1605	2292	2237	0.3780208600	0.0694246200	0.0058861076
Gdist:358378_583	3026	2956	0.5068979300	0.0717831200	0.0060328842
NS:68530_2435	7872	7729	0.5070144700	0.0717038700	0.0060662202
Gdist:677505_361	5053	4951	0.5056892300	0.0716651500	0.0061232545
Gdist:171492_5949	1162	1123	0.4413435800	0.0686219000	0.0062524611
NS:44697_2357	7464	7329	0.4529449500	0.0681998600	0.0062731717
Gdist:49299_1987	4013	3932	0.5082304900	0.0711105400	0.0063040531

NS:502686_4025	7553	7417	0.0655191500	0.0759106200	0.0063755174
Gdist:104582_1926	382	357	0.4883536000	0.0709804000	0.0063978429
Gdist:225064_1397	1642	1600	0.4304611300	0.0695340900	0.0064394267
GENE:90405_2929	6377	6260	0.4965921100	0.0711725500	0.0064716561
NS:90405_2929	8021	7877	0.4965921100	0.0711725500	0.0064716561
Gdist:38911_3951	3332	3260	0.4246540600	0.0696549300	0.0064922172
Gdist:250538_1638	1819	1772	0.3955672900	0.0679850300	0.0066097435
Gdist:542355_865	4266	4183	0.1775848800	0.0728886300	0.0067491169
Gdist:122225_1899	643	610	0.4905785400	0.0701904900	0.0068657143
Gdist:122233_871	644	611	0.4905785400	0.0701904900	0.0068657143
Gdist:412197_818	3584	3510	0.3877571500	0.0673730900	0.0070794129
NS:82343_3580	7981	7837	0.2976153500	0.0688653300	0.0071439618
NS:62508_3143	7766	7623	0.4300393900	0.0682749300	0.0071672684
Gdist:53268_1290	4214	4133	0.3263449400	0.0672760200	0.0072438326
Gdist:549795_7537	4328	4243	0.4389829600	0.0670276800	0.0073259812
Gdist:629798_2588	4720	4625	0.4507142800	0.0661822500	0.0073700090
Gdist:387072_2214	3310	3238	0.3604809200	0.0664995600	0.0073878962
NS:58607_7078	7709	7567	0.4449736300	0.0660507500	0.0075018247
MOEN:rs119054767	6617	6493	0.1962753900	0.0749627800	0.0075611531
Gdist:567183_405	4429	4342	0.4978455200	0.0690386800	0.0075917944
Gdist:311585_631	2501	2441	0.4821028400	0.0676942000	0.0078477171
Gdist:563611_457	4415	4329	0.4746347200	0.0665772300	0.0080921719
Gdist:227478_1391	1648	1606	0.5087152300	0.0678490000	0.0080999970
Gdist:79178_142	5494	5386	0.4645875300	0.0652418300	0.0081443675
Gdist:140485_2090	853	818	0.5095404400	0.0676334000	0.0081907724
Gdist:230877_295	1679	1635	0.3352321300	0.0661802300	0.0082137718
Gdist:250519_1303	1818	1771	0.3959950600	0.0654264900	0.0082517390
Gdist:250553_692	1820	1773	0.3959950600	0.0654264900	0.0082517390
Gdist:264081_3382	1945	1894	0.4720007800	0.0659490100	0.0082792843
Gdist:468631_2830	3909	3829	0.0932646400	0.0726210700	0.0083232016
GENE:468631_2830	6221	6105	0.0932646400	0.0726210700	0.0083232016

Gdist:214768_431	1563	1521	0.1712856700	0.0700417200	0.0085288187
NS:214768_431	6949	6823	0.1712856700	0.0700417200	0.0085288187
CAN:rs119056257	127	108	0.5058829900	0.0668773900	0.0088465228
Gdist:194176_490	1399	1359	0.1650090100	0.0697130000	0.0089297864
Gdist:337715_457	2783	2722	0.4381408800	0.0641142600	0.0093353275
Gdist:24797_1444	1792	1745	0.1708398000	0.0686116600	0.0094658800
Gdist:45577_491	3826	3748	0.5047958000	0.0658531800	0.0095911707
Gdist:439808_771	3759	3682	0.3297344500	0.0638756200	0.0097374069
Gdist:05268_7683	251	227	0.4130837500	0.0643192300	0.0098329266
Gdist:738165_418	5296	5189	0.4477790400	0.0625052400	0.0098351839

Table S3. List of 89 outliers used for analysis of 4 samples from the eastern Baltic stock (LAT, LIT, GDN, BOR).

SNP ID	ID	He	Obs. Fst	P-value
NS:286724_1728	7077	0.35460791	0.10816004	0.0000049104
Gdist:286656_9605	2190	0.39110864	0.10483655	0.0000126079
NS:66995_472	7834	0.43487509	0.10862613	0.0000507082
Gdist:343337_1473	2873	0.23694728	0.07826675	0.0002356582
Gdist:392627_1699	3388	0.17734481	0.08963327	0.0002618945
Gdist:731432_475	5247	0.29752783	0.08031841	0.0003020481
Gdist:266431_3965	1984	0.36657958	0.07922364	0.0003305230
CAN:rs119056004	80	0.42101736	0.09413603	0.0004195637
Gdist:57460_2290	4462	0.26487784	0.07407075	0.0005588475
Gdist:46602_1514	3899	0.04755439	0.0838624	0.0006078512
LD:411387_2719	6433	0.06639001	0.08739825	0.0008144813
Gdist:743040_2267	5339	0.16873071	0.07109013	0.0011590947
Gdist:297223_4120	2300	0.50551042	0.08393352	0.0012461787
GENE:432989_130	6210	0.38542691	0.06396874	0.0015415425

Gdist:320991_1112	2593	0.49950427	0.07719493	0.0016127438
Gdist:324226_148	2641	0.46816746	0.07453722	0.0016720805
Gdist:613313_244	4642	0.44822481	0.07966589	0.0018219729
NS:619203_761	7763	0.50031015	0.07478304	0.0019082803
Gdist:259369_862	1896	0.50315425	0.0745463	0.0020126468
Gdist:77627_601	5455	0.19959146	0.06588261	0.0020460013
Gdist:67626_1227	5037	0.15261692	0.06619222	0.0020683598
Gdist:325831_2074	2655	0.28923992	0.06448307	0.0021185740
Gdist:67152_1724	4974	0.45471352	0.07565683	0.0022263548
NS:404411_13389	7403	0.44843266	0.0764872	0.0022925962
Gdist:344017_785	2882	0.32683402	0.06515053	0.0025595849
NS:326236_13653	7191	0.45936962	0.07074916	0.0028291016
Gdist:53892_2073	4249	0.37032829	0.05911613	0.0030330585
Gdist:89516_1823	5717	0.34292691	0.06242049	0.0030672310
Gdist:140173_773	848	0.28263005	0.06069826	0.0030964093
Gdist:320502_666	2581	0.43681771	0.07157792	0.0031316496
Gdist:103543_1234	374	0.43396776	0.06999128	0.0034195431
MITO:872249_8578	6539	0.15731905	0.06101997	0.0034262552
Gdist:60766_572	4623	0.45062696	0.06967027	0.0035473861
Gdist:85953_634	5637	0.45733114	0.06814797	0.0035587902
GENE:398994_5000	6174	0.05684716	0.06608922	0.0037421854
Gdist:113467_3834	526	0.19816847	0.05796773	0.0041496891
NS:345568_1712	7229	0.50990648	0.06412873	0.0043327041
Gdist:651451_647	4819	0.30286362	0.05830946	0.0044218304
Gdist:735306_2067	5272	0.30832287	0.05837524	0.0044333433
Gdist:87655_1048	5670	0.47058078	0.06190289	0.0044339886
CAN:rs119056391	153	0.38485267	0.05451469	0.0045850067
Gdist:613469_515	4644	0.49372967	0.06225664	0.0046723845
Gdist:22409_144	1634	0.42122574	0.06304608	0.0047462457
Gdist:341010_437	2824	0.24288767	0.05739711	0.0048946929
Gdist:406445_130	3515	0.50245099	0.06176443	0.0050311076

GENE:417466_2181	6194	0.44838129	0.0643504	0.0050710657
Gdist:188350_1443	1305	0.50653945	0.061619	0.0051895190
Gdist:257132_468	1875	0.29505192	0.05605476	0.0052396686
Gdist:556415_5584	4372	0.04760913	0.05834046	0.0052698494
Gdist:134199_561	785	0.47921924	0.05979886	0.0053504120
Gdist:55097_312	4337	0.24979956	0.05648012	0.0055857855
Gdist:264402_506	1956	0.31419282	0.05532373	0.0058090730
Gdist:401491_3222	3454	0.3789713	0.05238387	0.0058695003
Gdist:595048_291	4566	0.37004358	0.05238295	0.0060997729
NS:179520_2574	6870	0.49378803	0.05880265	0.0062183055
LD:740049_1064	6501	0.13548292	0.05887255	0.0062618615
Gdist:188489_1180	1310	0.49078037	0.05848101	0.0062723059
Gdist:357812_7565	3013	0.50578039	0.05864045	0.0065393530
Gdist:122837_70	649	0.50939719	0.05863595	0.0065580510
Gdist:401305_286	3447	0.40787092	0.05564786	0.0067685467
Gdist:47096_1035	3920	0.46296446	0.05752083	0.0068566035
Gdist:98873_1526	5883	0.06572716	0.05938354	0.0068774982
GENE:67540_590	6314	0.06572716	0.05938354	0.0068774982
Gdist:210004_933	1539	0.30769434	0.053205	0.0070491107
Gdist:44006_1440	3763	0.06568611	0.05879567	0.0071713385
Gdist:694017_1476	5125	0.50451075	0.0573462	0.0072264807
GENE:671175_2957	6302	0.50792683	0.05731269	0.0072842872
Gdist:435985_6311	3738	0.40301984	0.05356823	0.0073812447
Gdist:174355_231	1190	0.2698814	0.05273971	0.0074112455
Gdist:67568_3745	5024	0.24291268	0.05338424	0.0074574466
FISH:rs119055837	175	0.26366194	0.05315661	0.0075557833
NS:292586_1455	7097	0.43902766	0.05738114	0.0077499171
Gdist:696194_2959	5134	0.50853994	0.05608199	0.0080308849
NS:144379_961	6797	0.43275841	0.05578491	0.0084418555
Gdist:243631_4426	1763	0.43909854	0.05592318	0.0085735780
Gdist:292075_1755	2264	0.17507505	0.05048545	0.0086068451

NS:648970_155	7799	0.33656645	0.05098177	0.0086132681
Gdist:185153_742	1278	0.50691829	0.05504221	0.0087466685
Gdist:135342_1135	794	0.46550143	0.05393812	0.0087876490
Gdist:335642_427	2765	0.40260072	0.05149146	0.0088104631
Gdist:61046_1277	4636	0.36408253	0.0488616	0.0088279692
Gdist:357647_822	3008	0.30831945	0.05064495	0.0088651188
Gdist:114711_678	546	0.50965397	0.05458043	0.0090462779
Gdist:822912_1679	5571	0.2638415	0.05121596	0.0091224505
Gdist:342896_5486	2858	0.44712887	0.05538497	0.0092522899
NS:145884_1491	6802	0.4865851	0.05373139	0.0092622342
Gdist:104700_673	386	0.45598919	0.05455924	0.0094111152
NS:29894_959	7109	0.24274242	0.0510252	0.0094129016
Gdist:184304_1970	1275	0.24915872	0.05105579	0.0096255585

Table S4. List of 76 outlier SNPs used for analysis of 3 eastern Baltic samples.

SNP ID	ID		He	Obs. Fst	P-value
CAN:rs119056004	80	64	0.406227	0.123789	0.0000308805
Gdist:297223_4120	2300	2245	0.507686	0.111472	0.0001575129
Gdist:324226_148	2641	2581	0.467876	0.106202	0.0002124589
Gdist:60766_572	4623	4530	0.458748	0.105787	0.0003156543
Gdist:46602_1514	3899	3819	0.059797	0.089293	0.0009723922
GENE:417466_2181	6194	6079	0.445264	0.093527	0.0010944222
LD:411387_2719	6433	6315	0.08308	0.088416	0.0012337708
Gdist:22409_144	1634	1592	0.412345	0.088771	0.0012746264
GENE:432989_130	6210	6094	0.375607	0.090809	0.0012776765
NS:404411_13389	7403	7270	0.427914	0.086781	0.0014001549
Gdist:320991_1112	2593	2533	0.513926	0.091862	0.0014025374

Gdist:53892_2073	4249	4166	0.38212	0.085869	0.0015247139
NS:179520_2574	6870	6746	0.502269	0.08906	0.0017528866
Gdist:98873_1526	5883	5772	0.071238	0.082292	0.0019277742
GENE:67540_590	6314	6197	0.071238	0.082292	0.0019277742
Gdist:435985_6311	3738	3662	0.405607	0.082217	0.0019840900
Gdist:335642_427	2765	2704	0.411178	0.0799	0.0021929564
Gdist:335655_1240	2766	2705	0.411178	0.0799	0.0021929564
NS:648970_155	7799	7656	0.340252	0.080026	0.0024408519
Gdist:342896_5486	2858	2795	0.44269	0.080245	0.0024588942
Gdist:651451_647	4819	4720	0.312095	0.085643	0.0025734905
MITO:872249_8578	6539	6416	0.175741	0.081488	0.0027229446
Gdist:696194_2959	5134	5032	0.514747	0.082527	0.0028103666
Gdist:334772_405	2750	2689	0.437482	0.076725	0.0028363840
Gdist:114711_678	546	515	0.51705	0.082222	0.0028980130
Gdist:135342_1135	794	760	0.477876	0.077112	0.0032825763
FISH:rs119055837	175	151	0.244514	0.076449	0.0032913527
GENE:671175_2957	6302	6185	0.516517	0.080152	0.0033200779
Gdist:47096_1035	3920	3839	0.47757	0.07681	0.0033377932
Gdist:323213_4530	2621	2561	0.402711	0.072081	0.0036610490
NS:280890_687	7056	6927	0.243556	0.072815	0.0041926847
Gdist:148382_1003	949	913	0.398673	0.069668	0.0042495022
Gdist:269670_678	2016	1964	0.398673	0.069668	0.0042495022
Gdist:357647_822	3008	2939	0.303606	0.078924	0.0043470407
Gdist:139020_213	835	800	0.252608	0.072237	0.0045981414
NS:139020_213	6790	6666	0.252608	0.072237	0.0045981414
NS:385726_883	7344	7211	0.392251	0.068239	0.0046603049
Gdist:324295_3574	2643	2583	0.093484	0.071886	0.0047574699
MOEN:rs119054653	6582	6458	0.093484	0.071886	0.0047574699
Gdist:327490_108	2674	2613	0.500598	0.074398	0.0047959171
Gdist:268362_7093	2009	1957	0.346815	0.068532	0.0050137530

NS:182913_1266	6878	6754	0.489235	0.072496	0.0051265536
Gdist:97162_2313	5855	5744	0.515351	0.073277	0.0051631672
NS:619203_761	7763	7620	0.488142	0.071614	0.0053711475
NS:102328_1836	6671	6547	0.470842	0.06898	0.0053924907
Gdist:735306_2067	5272	5165	0.332395	0.069815	0.0054494057
Gdist:19135_1654	1348	1309	0.324116	0.06962	0.0060297230
Gdist:292075_1755	2264	2209	0.187077	0.070846	0.0060362005
NS:40437_8621	7402	7269	0.360415	0.065135	0.0061359580
Gdist:77627_601	5455	5347	0.15736	0.067011	0.0061949752
Gdist:02700_1649	212	188	0.504996	0.07032	0.0061953316
GENE:398994_5000	6174	6059	0.07109	0.066501	0.0063606234
NS:145884_1491	6802	6678	0.50046	0.068688	0.0067826959
Gdist:311243_2031	2495	2435	0.514416	0.068549	0.0069467357
Gdist:595326_441	4570	4479	0.11544	0.064402	0.0076118301
Gdist:61046_1277	4636	4542	0.38507	0.060619	0.0078131735
CAN:rs119055580	21	14	0.323694	0.065011	0.0081113067
Gdist:188350_1443	1305	1266	0.514232	0.065913	0.0081924033
Gdist:40414_5390	3489	3417	0.115598	0.063428	0.0082112522
Gdist:360398_615	3055	2984	0.397036	0.06006	0.0083393324
Gdist:160789_2454	1068	1029	0.513221	0.065084	0.0086007929
Gdist:243631_4426	1763	1716	0.459957	0.06091	0.0086919509
Gdist:391025_2403	3352	3280	0.302007	0.067567	0.0087050300
Gdist:55097_312	4337	4252	0.215636	0.064636	0.0087436991
Gdist:184304_1970	1275	1236	0.268866	0.064914	0.0088437533
Gdist:409327_360	3535	3461	0.359487	0.059497	0.0092428619
NS:88015_4153	8006	7862	0.196664	0.063335	0.0092630862
Gdist:530961_8421	4200	4119	0.508804	0.063752	0.0093078153
Gdist:822912_1679	5571	5463	0.234118	0.060321	0.0094068904
Gdist:95695_2959	5812	5701	0.507475	0.063365	0.0095160295
Gdist:87971_586	5679	5569	0.512885	0.063334	0.0095850244
GENE:432988_180	6209	6093	0.242304	0.05984	0.0096329311

Gdist:87655_1048	5670	5560	0.489151	0.061847	0.0096576683
Gdist:243678_594	1764	1717	0.493285	0.062043	0.0097785909
Gdist:358773_3819	3033	2963	0.396817	0.057787	0.0098461185
Gdist:04769_193	236	212	0.294023	0.066325	0.0098524661

Table S5. Outliers loci. SNP individual ID, expected heterozygosity, observed FST, p value, linkage map position and gene ontology analysis results generated from databeses: Ensembl, NCBI (blastn) and UniProt.

SNP ID	ID	He	Obs. Fst	*p*-value	dbSNP	Chromosome	Possition in scaffold/ contig	Ensembl	Ensembl name	NCBI (blastn)	UniProt / Function
NS:286724_1728	7077	0.35460791	0.10816004	0.00000	17491124	LG13	17491124	NA	NA	Gadus morhua ADP-ribosylation factor 4-like (LOC115557017), mRNA	Intracellular protein transport
Gdist:286656_9605	2190	0.39110864	0.10483655	0.00001	17839335	LG13	17839335	DNA-damage inducible protein 2	ddi2	NA	NA
NS:66995_472	7834	0.43487509	0.10862613	0.00005	16214527	LG13	16214527	NA	NA	Gadus morhua protein-glutamine gamma-glutamyltransferase 6-like (LOC115557882), mRNA	Metal ion binding
Gdist:343337_1473	2873	0.23694728	0.07826675	0.00024	8906878	LG20	8906878	NA	NA	NA	NA
Gdist:392627_1699	3388	0.17734481	0.08963327	0.00026	22695718	LG03	22695718	inositol polyphosphate-5-phosphatase B	inpp5b	NA	NA
Gdist:731432_475	5247	0.29752783	0.08031841	0.00030	5260546	LG03	5260546	NA	NA	NA	NA
Gdist:266431_3965	1984	0.36657958	0.07922364	0.00033	17475408	LG18	17475408	ATP-binding cassette. sub-family C (CFTR/MRP), member 3	abcc3	NA	NA
CAN:rs119056004	80	0.42101736	0.09413603	0.00042	5946360	LG05	5946360	NA	NA	Gadus morhua solute carrier family 66 member 3 (slc66a3), mRNA	NA
Gdist:57460_2290	4462	0.26487784	0.07407075	0.00056	12035233	LG11	12035233	dpy-19-like 1. like (H. sapiens)	dpy19l1l	NA	NA
Gdist:46602_1514	3899	0.04755439	0.0838624	0.00061	7089547	LG05	7089547	neuregulin 2a	nrg2a	NA	NA
LD:411387_2719	6433	0.06639001	0.08739825	0.00081	5886411	LG06	5886411	E1A binding protein p400	ep400	NA	NA
Gdist:743040_2267	5339	0.16873071	0.07109013	0.00116	13313059	LG04	13313059	NA	NA	NA	NA
Gdist:297223_4120	2300	0.50551042	0.08393352	0.00125	19698364	LG14	19698364	WT1 transcription factor b	wt1b	NA	NA
GENE:432989_130	6210	0.38542691	0.06396874	0.00154	20272231	LG21	20272231	meprin A. alpha (PABA peptide hydrolase), tandem duplicate 2	mep1a.2	Gadus morhua meprin A subunit alpha-like (LOC115534751), mRNA	NA
Gdist:320991_1112	2593	0.49950427	0.07719493	0.00161	15389618	LG03	15389618	centlein. centrosomal protein	cntln	NA	NA
Gdist:324226_148	2641	0.46816746	0.07453722	0.00167	5333731	LG18	5333731	NA	NA	NA	NA
Gdist:613313_244	4642	0.44822481	0.07966589	0.00182	25686448	LG08	25686448	NA	NA	NA	NA
NS:619203_761	7763	0.50031015	0.07478304	0.00191	1315076	LG22	1315076	NA	NA	Gadus morhua shugoshin 1-like (LOC115535338). transcript variant X2, mRNA	NA
Gdist:259369_862	1896	0.50315425	0.0745463	0.00201	2412326	LG02	2412326	NA	NA	NA	NA
Gdist:77627_601	5455	0.19959146	0.06588261	0.00205	9905369	LG18	9905369	transformation/transcription domain-associated protein	trrap	NA	NA
Gdist:67626_1227	5037	0.15261692	0.06619222	0.00207	17870661	LG14	17870661	NA	NA	NA	NA

Gdist:325831_2074	2655	0.28923992	0.06448307	0.00212	24258491	LG01	24258491	fibrillin 2b	fbn2b	Gadus morhua suppressor of tumorigenicity 14 protein homolog (LOC115550873), mRNA	Degrades extracellular matrix. Exhibits trypsin-like activity as defined by cleavage of synthetic substrates with Arg or Lys as the P1 site
Gdist:67152_1724	4974	0.45471352	0.07565683	0.00223	15431308	LG13	15431308	NA	NA	NA	NA
NS:404411_13389	7403	0.44843266	0.0764872	0.00229	19691778	LG02	19691778	major facilitator superfamily domain containing 11	mfsd11	Gadus morhua methyltransferase like 23 (mettl23). transcript variant X2, mRNA	Probable methyltransferase
Gdist:344017_785	2882	0.32683402	0.06515053	0.00256	11779152	LG23	11779152	NA	NA	NA	NA
NS:326236_13653	7191	0.45936962	0.07074916	0.00283	13420604	LG01	13420604	glutaminyl-tRNA synthetase	qars	Gadus morhua glutaminyl-tRNA synthetase 1 (qars1), mRNA	NA
Gdist:53892_2073	4249	0.37032829	0.05911613	0.00303	10251654	LG13	10251654	potassium voltage-gated channel. shaker-related subfamily, beta member 2 a	kcnab2a	NA	NA
Gdist:89516_1823	5717	0.34292691	0.06242049	0.00307	8102134	LG22	8102134	deleted in azoospermia-like	dazl	NA	NA
Gdist:140173_773	848	0.28263005	0.06069826	0.00310	29253465	LG11	29253465	pescadillo	pes	NA	NA
Gdist:320502_666	2581	0.43681771	0.07157792	0.00313	20304330	LG17	20304330	NA	NA	NA	NA
Gdist:103543_1234	374	0.43396776	0.06999128	0.00342	30462239	LG12	30462239	NA	NA	Gadus morhua niban apoptosis regulator 1 (niban1), mRNA	Regulates phosphorylation of a number of proteins involved in translation regulation including EIF2A. EIF4EBP1 and RPS6KB1. May be involved in the endoplasmic reticulum stress response
MITO:872249_8578	6539	0.15731905	0.06101997	0.00343	NA	NA	NA	NA	NA	NA	NA
Gdist:60766_572	4623	0.45062696	0.06967027	0.00355	12878865	LG22	12878865	Ras protein specific guanine nucleotide releasing factor 1	RASGRF1	NA	NA
Gdist:85953_634	5637	0.45733114	0.06814797	0.00356	19897908	LG20	19897908	coiled-coil domain containing 148	CCDC148	NA	NA
GENE:398994_5000	6174	0.05684716	0.06608922	0.00374	8442333	LG21	8442333	estrogen receptor 2a	esr2a	NA	NA
Gdist:113467_3834	526	0.19816847	0.05796773	0.00415	11919888	LG13	11919888	importin 9	ipo9	NA	NA
NS:345568_1712	7229	0.50990648	0.06412873	0.00433	28517132	LG11	28517132	cholesterol 25-hydroxylase like 3	ch25ha	Gadus morhua cholesterol 25-hydroxylase-like protein (LOC115554742), transcript variant X2, mRNA	Catalyzes the formation of 25-hydroxycholesterol from cholesterol. leading to repress cholesterol biosynthetic enzymes
Gdist:651451_647	4819	0.30286362	0.05830946	0.00442	7273406	LG10	7273406	coagulation factor VIII. procoagulant component	f8	Gadus morhua coagulation factor VIII-like (LOC115552590), mRNA	Platelet activation
Gdist:735306_2067	5272	0.30832287	0.05837524	0.00443	26532995	LG08	26532995	NA	NA	NA	NA
Gdist:87655_1048	5670	0.47058078	0.06190289	0.00443	13984681	LG10	13984681	catenin delta 1	CTNND1	NA	NA
CAN:rs119056391	153	0.38485267	0.05451469	0.00459	24982620	LG15	24982620	NA	NA	Gadus morhua adenosine kinase-like (LOC115559959), transcript variant X2, mRNA	NA
Gdist:613469_515	4644	0.49372967	0.06225664	0.00467	22671699	LG05	22671699	NA	NA	NA	NA
Gdist:22409_144	1634	0.42122574	0.06304608	0.00475	3971960	LG23	3971960	ADAM metallopeptidase domain 15	adam15	NA	NA
Gdist:341010_437	2824	0.24288767	0.05739711	0.00489	7141194	LG12	7141194	NA	NA	NA	NA
Gdist:406445_130	3515	0.50245099	0.06176443	0.00503	5840807	LG01	5840807	NA	NA	NA	NA

GENE:417466_2181	6194	0.44838129	0.0643504	0.00507	22469616	LG20	22469616	NA	NA	NA	NA
Gdist:188350_1443	1305	0.50653945	0.061619	0.00519	29407321	LG11	29407321	ubiquitin protein ligase E3B	ube3b	Gadus morhua ubiquitin protein ligase E3B (ube3b), mRNA	E3 ubiquitin-protein ligase which accepts ubiquitin from an E2 ubiquitin-conjugating enzyme in the form of a thioester and then directly transfers the ubiquitin to targeted substrates
Gdist:257132_468	1875	0.29505192	0.05605476	0.00524	17401069	LG03	17401069	NA	NA	NA	NA
Gdist:556415_5584	4372	0.04760913	0.05834046	0.00527	3738092	LG05	3738092	solute carrier family 18 member B1	slc18b1	NA	NA
Gdist:134199_561	785	0.47921924	0.05979886	0.00535	15058842	LG17	15058842	NA	NA	Gadus morhua RNA polymerase II subunit A (polr2a), mRNA	DNA-dependent RNA polymerase catalyzes the transcription of DNA into RNA using the four ribonucleoside triphosphates as substrates
Gdist:55097_312	4337	0.24979956	0.05648012	0.00559	8003390	LG05	8003390	discs. large (Drosophila) homolog-associated protein 2a	dlgap2a	NA	
Gdist:264402_506	1956	0.31419282	0.05532373	0.00581	10402791	LG07	10402791	NA	NA	Gadus morhua dual specificity protein kinase CLK4-like (LOC115547459). transcript variant X5, mRNA	
Gdist:401491_3222	3454	0.3789713	0.05238387	0.00587	9989784	LG23	9989784	elastin microfibril interfacer 2b	emilin2.2	Gadus morhua elastin microfibril interfacer 2 (emilin2), mRNA	Responsible for anchoring smooth muscle cells to elastic fibers. and may be involved not only in the formation of the elastic fiber. but also in the processes that regulate vessel assembly
Gdist:595048_291	4566	0.37004358	0.05238295	0.00610	20188874	LG22	20188874	NA	NA	NA	NA
NS:179520_2574	6870	0.49378803	0.05880265	0.00622	10982198	LG08	10982198	NA	NA	Gadus morhua zonadhesin-like (LOC115548780). transcript variant X5, mRNA	NA
LD:740049_1064	6501	0.13548292	0.05887255	0.00626	14571999	LG15	14571999	protein tyrosine phosphatase receptor type Eb	ptpreb	NA	NA
Gdist:188489_1180	1310	0.49078037	0.05848101	0.00627	20610017	LG18	20610017	collagen. type I. alpha 1b	col1a1b	Gadus morhua collagen alpha-1(I) chain-like (LOC115530900), mRNA	NA
Gdist:357812_7565	3013	0.50578039	0.05864045	0.00654	10050765	LG01	10050765	G patch domain and KOW motifs	gpkow	Gadus morhua G-patch domain and KOW motifs (gpkow), mRNA	RNA-binding protein involved in pre-mRNA splicing.
Gdist:122837_70	649	0.50939719	0.05863595	0.00656	9946387	LG02	9946387	kinesin family member 13A	kif13a	NA	NA
Gdist:401305_286	3447	0.40787092	0.05564786	0.00677	10813354	LG23	10813354	Gadus morhua Rho guanine nucleotide exchange factor 4 (arhgef4). transcript variant X3, mRNA		Gadus morhua Rho guanine nucleotide exchange factor 4 (arhgef4). transcript variant X3, mRNA	Acts as guanine nucleotide exchange factor (GEF) for RHOA. RAC1 and CDC42 GTPases.
Gdist:47096_1035	3920	0.46296446	0.05752083	0.00686	16534366	LG01	16534366	NA	NA	Gadus morhua SAM and HD domain containing deoxynucleoside triphosphate triphosphohydrolase 1 (samhd1), transcript variant X2, mRNA	Acts both as a host restriction factor involved in defense response to virus and as a regulator of DNA end resection at stalled replication forks
Gdist:98873_1526	5883	0.06572716	0.05938354	0.00688	432740	LG06	432740	NA	NA	NA	NA
GENE:67540_590	6314	0.06572716	0.05938354	0.00688	17409710	LG14	17409710	NA	NA	NA	NA
Gdist:210004_933	1539	0.30769434	0.053205	0.00705	15551167	LG09	15551167	NA	NA	Gadus morhua mannosidase alpha class 2A member 2 (man2a2), transcript variant X6, mRNA	Catalyzes the first committed step in the biosynthesis of complex N-glycans

Gdist:44006_1440	3763	0.06568611	0.05879567	0.00717	3586886	LG23	3586886	NA	NA	NA	NA
Gdist:694017_1476	5125	0.50451075	0.0573462	0.00723	7626099	LG02	7626099	magnesium transporter MRS2	mrs2	NA	NA
GENE:671175_2957	6302	0.50792683	0.05731269	0.00728	11900490	LG21	11900490	thyroid stimulating hormone receptor	tshr	Gadus morhua thyrotropin receptor-like (LOC115534392) mRNA	Combining with thyrotropin-releasing hormone to initiate a change in cell activity
Gdist:435985_6311	3738	0.40301984	0.05356823	0.00738	17225863	LG11	17225863	activating signal cointegrator 1 complex subunit 3	ascc3	NA	NA
Gdist:174355_231	1190	0.2698814	0.05273971	0.00741	19744678	LG22	19744678	syndecan 2	sdc2	NA	NA
Gdist:67568_3745	5024	0.24291268	0.05338424	0.00746	17555305	LG14	17555305	NA	NA	NA	NA
FISH:rs119055837	175	0.26366194	0.05315661	0.00756	6540478	LG11	6540478	NA	NA	Gadus morhua aryl hydrocarbon receptor nuclear translocator-like (LOC115553933). transcript variant X3, mRNA	Forms a core component of the circadian clock.
NS:292586_1455	7097	0.43902766	0.05738114	0.00775	2156785	LG16	2156785	gem (nuclear organelle) associated protein 4	gemin4	Gadus morhua gem nuclear organelle associated protein 4 (gemin4). transcript variant X2, mRNA	The SMN complex plays a catalyst role in the assembly of small nuclear ribonucleoproteins (snRNPs). the building blocks of the spliceosome
Gdist:696194_2959	5134	0.50853994	0.05608199	0.00803	5065416	LG05	5065416	NA	NA	NA	NA
NS:144379_961	6797	0.43275841	0.05578491	0.00844	16847984	LG08	16847984	tocopherol (alpha) transfer protein	ttpa	Gadus morhua alpha tocopherol transfer protein (ttpa), mRNA	Binds alpha-tocopherol. enhances its transfer between separate membranes. and stimulates its release from liver cells
Gdist:243631_4426	1763	0.43909854	0.05592318	0.00857	18663211	LG05	18663211	NA	NA	NA	NA
Gdist:292075_1755	2264	0.17507505	0.05048545	0.00861	21776842	LG22	21776842	NA	NA	NA	NA
NS:648970_155	7799	0.33656645	0.05098177	0.00861	27394216	LG14	27394216	NA	NA	NA	NA
Gdist:185153_742	1278	0.50691829	0.05504221	0.00875	20496051	LG21	20496051	NA	NA	NA	NA
Gdist:135342_1135	794	0.46550143	0.05393812	0.00879	21981629	LG19	21981629	plexin A4	plxna4	NA	NA
Gdist:335642_427	2765	0.40260072	0.05149146	0.00881	33834916	LG16	33834916	NA	NA	NA	NA
Gdist:61046_1277	4636	0.36408253	0.0488616	0.00883	14125694	LG22	14125694	NA	NA	NA	NA
Gdist:357647_822	3008	0.30831945	0.05064495	0.00887	9162966	LG01	9162966	NA	NA	NA	NA
Gdist:114711_678	546	0.50965397	0.05458043	0.00905	11269262	LG01	11269262	NA	NA	NA	NA
Gdist:822912_1679	5571	0.2638415	0.05121596	0.00912	24588557	LG06	24588557	NA	NA	NA	NA
Gdist:342896_5486	2858	0.44712887	0.05538497	0.00925	6799878	LG20	6799878	solute carrier family 4 member 3	slc4a3	Gadus morhua anion exchange protein 3-like (LOC115532893). transcript variant X5. mRNA	Plasma membrane anion exchange protein of wide distribution
NS:145884_1491	6802	0.4865851	0.05373139	0.00926	2054356	LG12	2054356	deoxythymidylate kinase (thymidylate kinase)	dtymk	Gadus morhua deoxythymidylate kinase (dtymk), mRNA	Catalyzes the conversion of dTMP to dTDP
Gdist:104700_673	386	0.45598919	0.05455924	0.00941	18762768	LG02	18762768	NA	NA	NA	NA
NS:29894_959	7109	0.24274242	0.0510252	0.00941	NA	NA	NA	NA	NA	NA	NA
Gdist:184304_1970	1275	0.24915872	0.05105579	0.00963	22743385	LG08	22743385	NA	NA	Gadus morhua eukaryotic translation initiation factor 4 gamma 1-like (LOC115548395). transcript variant X10, mRNA	NA
